# Single-cell dissection reveals the role of aggrephagy patterns in tumor microenvironment components aiding predicting prognosis and immunotherapy on lung adenocarcinoma

**DOI:** 10.18632/aging.205306

**Published:** 2023-12-13

**Authors:** Xinti Sun, Fei Meng, Minyu Nong, Hao Fang, Chenglu Lu, Yan Wang, Peng Zhang

**Affiliations:** 1 Department of Cardiothoracic Surgery, Tianjin Medical University General Hospital, Tianjin 300052, China; 2 School of Clinical Medicine, Youjiang Medical University for Nationalities, Baise, Guangxi, China; 3 Department of Pathology, Tianjin Medical University Cancer Institute and Hospital, Tianjin, China

**Keywords:** aggrephagy, lung cancer, bioinformatics, tumor microenvironment, scRNA

## Abstract

Background: Lung adenocarcinoma (LUAD) is one of the leading malignant cancers. Aggrephagy plays a critical role in key genetic events for various cancers; yet, how aggrephagy functions within the tumor microenvironment (TME) in LUAD remains to be elucidated.

Methods: In this study, by sequential non-negative matrix factorization (NMF) algorithm, pseudotime analysis, cell-cell interaction analysis, and SCENIC analysis, we have shown that aggrephagy genes demonstrated various patterns among different cell types in LUAD TME. LUAD and Immunotherapy cohorts from public repository were used to determine the prognosis and immune response of aggrephagy TME subtypes. The aggrephagy-deprived prognostic score (ADPS) was quantified based on machine learning algorithms.

Results: The cancer-associated fibroblasts (CAFs), tumor-associated macrophages (TAMs), and CD8+ T cells have various aggrephagy patterns, which enhance the intensity of intercellular communication and transcription factor activation. Furthermore, based on the signatures of the newly defined aggrephagy cell subtypes and expression profiles of large cohorts in LUAD patients, we determine that DYNC1I2+CAF-C1, DYNLL1+CAF-C2, PARK7+CAF-C3, VIM+Mac-C1, PARK7+Mac-C2, VIM+CD8+T_cells-C1, UBA52+CD8+T_cells-C2, TUBA4A+CD8+T_ cells-C3, and TUBA1A+CD8+T_cells-C4 are crucial prognostic factors for LUAD patients. The developed ADPS could predict survival outcomes and immunotherapeutic response across ten cohorts (*n* = 1838), and patients with low ADPS owned a better prognosis, lower genomic alterations, and are more sensitive to immunotherapy. Meanwhile, based on PRISM, CTRP, and CMAP databases, PLK inhibitor BI-2536, may be a potential agent for patients with high ADPS.

Conclusions: Taken together, our novel and systematic single-cell analysis has revealed the unique role of aggrephagy in remodeling the TME of LUAD. As a newly demonstrated biomarker, the ADPS facilitates the clinical management and individualized treatment of LUAD.

## INTRODUCTION

Non-small cell lung cancer (NSCLC) is a malignancy with a notable prevalence and mortality rate, wherein the lung adenocarcinoma (LUAD) represents the foremost prevalent pathological subclass [1]. The amassing of molecular insights through burgeoning technologies has facilitated the development of targeted therapeutics alongside traditional interventions, such as surgery and chemotherapy. The 5-year relative survival rate of LUAD patients, at 21%, mirrors the difficulties encountered in managing this aggressive malignancy, frequently detected at an advanced stage, whereby the disease has already metastasized to other regions of the body [2]. The difficulty in treating LUAD is often due to its resistance to traditional chemotherapy and radiation therapy, which could lead to different individual therapeutic responses and contribute to its poor survival rate. Tumor heterogeneity, including characterized by diverse TME patterns as well as cancer cell types, represents a significant contributor to reduced response rates and drug resistance [3]. Thankfully, advances in molecular biology, genomics, and proteomics might help unravel the complexity as well as heterogeneity at the molecular level, leading to new individualized strategies for LUAD treatment.

Autophagy represents a highly conserved eukaryotic cellular recycling process that plays an indispensable role in the degradation of cytoplasmic organelles, proteins, as well as macromolecules, essential for the survival and maintenance of cells [4]. Significantly, autophagy serves as a sustainable source of biomolecules and energy to maintain homeostasis under stressful conditions, including those encountered within the tumor microenvironment [5]. Misfolded proteins accumulate in cells to form protein aggregates, which interfere with the normal physiological activities of cells and cause various human diseases [6, 7]. Aggrephagy is a specific clearance pathway for intracellular protein aggregates, which is a type of selective autophagy. It undertakes most of the tasks involved in degrading protein aggregates and plays a decisive role in the abnormal protein quality control system. This pathway has potential applications in the treatment of various diseases, like neurodegenerative diseases, muscular atrophy, and cancer [8]. Previous studies have indicated that protein misfolding and aggregation can impact the function of the p53 protein in cancer. When the p53 protein is mutated or aggregated, it can lose its functionality, leading to tumor progression [9–11]. Consequently, comprehending the mechanisms of aggrephagy has arisen as a promising approach for targeted therapy as well as holds potential for further research in drug development. Single-cell transcriptomics has uncovered the intricate intercellular crosstalk between diverse subtypes of cells in the TME and cancer cells, revealing a complex network of intercellular signaling pathways that regulate tumor growth and progression [12]. The TME consists of a diverse range of cellular components, including CAFs, TAMs, T cells, and tumor cells. It is defined by a tumor-promoting and immunosuppressive phenotype, characterized by complex intercellular signaling networks that regulate the progression and growth of the tumor [13]. Meanwhile, TME has long been known as a nutrient-depleted environment, study indicated that the autophagy of cancer cells rescued itself from T cell-mediated cytotoxicity by blocking cytokine-induced apoptosis [14]. Zhao et al. found that autophagy loss impedes CAFs activation via downregulation proline biosynthesis [15]. Additionally, wang et al. discovered that elevated TUBA1A, a classical aggrephagy markers, lead the worse clinical outcomes of gastric cancer patients, and be associated with the infiltration of macrophages in the TME [16]. PARK7, alternatively referred to as DJ-1, is overexpressed in a substantial 86% of individuals diagnosed with NSCLC. 72.2% predominantly exhibit PARK7 expression in the cytoplasm, and the heightened expression of this gene is strongly correlated with unfavorable clinical outcomes, including increased risk of recurrence and reduced overall survival rates for LUAD patients [17, 18]. However, there has been limited research investigating the cell-cell interactions between TME cell subtypes and prognosis associated with aggrephagy modification in LUAD.

We explored the influence of aggrephagy on the main TME cells based on LUAD single-cell RNA sequencing data. Through NMF analysis of 44 aggrephagy genes, as previously described [19], it was discovered that distinct expression patterns of aggrephagy mRNA in various subpopulations of LUAD TME cells demonstrated extensive and diverse intercellular communication with epithelial cells, and associated with different immune characteristics, metabolic pathways, as well as transcription characteristics. Moreover, upon integration with the bulk RNA-seq data of sizable LUAD patient cohorts, we substantiated that multiple aggrephagy cell subtypes exerted a substantial influence on both the prognosis and response to immune checkpoint blockade (ICB) therapy. Based on this, we created and multicenter assessed a 32-gene combined ADPS via machine learning algorithms. ADPS has shown strong predictive ability for survival outcomes, immunotherapy response, and drug efficacy in multiple multicenter cohorts. To our knowledge, this extensive single-cell analysis is the first to unveil the potential role of aggrephagy mRNA in mediating intercellular communication between TME cells and tumor cells, thus impeding the progression of LUAD. Furthermore, our findings provide crucial insights for early detection, prognostic assessment, risk stratification, and personalized therapeutic interventions in clinical settings.

## MATERIALS AND METHODS

### Data acquisition

scRNA-seq data were obtained from the GEO database under accession number: GSE149655 [20]. LUAD bulk RNA-seq data including clinical traits were acquired from the GDC portal of TCGA and GEO databases: TCGA-LUAD, GSE3141, GSE31210, GSE37745, GSE50081, and GSE68465. The six datasets of 1512 patients were integrated and batch effects were adjusted by the Combat algorithm using the “sva” package [21]. Normal lung tissue data were available from the GTEx and TCGA databases. Two datasets treated with PD-(L)1 and containing clinical traits were downloaded: IMvigor210 [22], and GSE78220 [23]. Supplementary Table 1 summarizes the data sources and details of this study. A total of 44 aggrephagy genes were downloaded from https://www.gsea-msigdb.org/gsea/msigdb/cards/REACTOME_AGGREPHAGY.

### scRNA-seq data process

The normalization, integration, dimension reduction, and clustering were performed stepwise using the Seurat pipeline with the R package “Seurat” [24]. Cell annotation was performed by referring to common tumor microenvironment cell markers, published studies [25, 26], the CellMarker website (http://xteam.xbio.top/CellMarker/), and the PanglaoDB website (https://panglaodb.se/). Normalization data from the Seurat object were analyzed by single-cell NMF based on aggrephagy gene expression [27]. Cells that expressed no aggrephagy-related genes and aggrephagy-related genes that had no expression in all cell types were removed during the analysis. The NMF method was set to snmf/r and a maximum of ten clusters was used. The FindAllMarkers function was employed to identify the markers of each NMF cluster for every cell type in LUAD. Clusters with aggrephagy genes with log2 (fold change) exceeding 1.0 were termed (“Gene + Cell type”). Clusters with no characteristics of aggrephagy genes were termed “Non-Aggrephagy-Cell type”. Clusters with characteristics aggrephagy genes with log2 (fold change) less than 1 were termed “Unclear-Cell type”.

### Pseudotime trajectory, cell-cell interaction analysis, and transcription factor analysis

To explore the correlation between aggrephagy genes and cell pseudotime trajectories, we utilized the R package “Monocle2” to analyze scRNA data for all cell types in LUAD [28, 29]. In short, the Monocle object underwent size factor and dispersion estimation, followed by the identification of highly variable features. Afterward, dimensionality reduction was performed and cell ordering was carried out for the purpose of pseudotime visualization. Besides, cell-cell interactions were conducted using “CellChat”, an R package that identifies and quantifies intercellular communication between different cell types within a single-cell dataset [30]. Secreted signaling in humans was included in the cell-cell interaction analysis [31]. We utilized the “pySCENIC” package, which is a Python-based implementation of the SCENIC pipeline, to explore the transcription factor (TF) gene regulatory network in LUAD [32]. The scRNA-seq data of LUAD were subjected to analysis using two gene-motif rankings (hg19-tss-centered-10 kb and hg19-500 bp-upstream) obtained from the RcisTarget database, with the aim of detecting transcription start sites (TSS) and gene regulatory networks. TFs with a Benjamini-Hochberg false discovery rate (BH-FDR) <0.05 were selected for further investigation.

### Gene set scoring

The package “GSVA” was utilized to perform single-sample gene set enrichment analysis (ssGSEA) for gene set scoring in bulk RNA sequencing data [33, 34]. GSVA was also employed to assess the previously established CAF-subtype in the single-cell RNA sequencing data [35]. The “AddModuleScore” function was utilized to evaluate the expression of M1-like/M2-like polarization markers derived from published studies [36]. The metabolic scores of different aggrephagy cell subtypes were calculated using “scMetabolism” package [37].

### Survival analyses and ICB response prediction

We utilized the “GSVA” package to calculate the gene signature scores of the aggrephagy cell subtypes across all LUAD cohorts. The relationship between aggrephagy-related NMF signatures and patients’ prognosis was explored using the log-rank test and Cox proportional hazard regression. The “survminer” package was applied to plot Kaplan-Meier curves and determine the cutoff values of different NMF cell signatures in the different LUAD cohorts. Furthermore, we utilized the Tumor Immune Dysfunction and Exclusion (TIDE) algorithm available at http://tide.dfci.harvard.edu/ to obtain TIDE scores, which enabled us to predict the clinical response to ICB in LUAD cohorts. Prognosis-associated genes were identified by performing univariate Cox regression analysis with a significance threshold of *P* < 0.05 on the aforementioned aggrephagy cell subtypes that were associated with prognosis. To compress the gene number and identify the variables that have the greatest impact on the target variable, we employed a two-step approach to analyze the data, beginning with a LASSO Cox regression analysis to shrink the coefficient estimates. Subsequently, a multivariate Cox regression analysis with stepwise regression method was performed to identify the most significant predictors of the prognosis [38]. Based on the results of the multivariate Cox model, ADPS was calculated using the following formula: ADPS = Σβ × Expi. Having established that *i* denotes a gene in the ADPS, *expi* represents the expression level of gene *i*, as well as *βi* stands for the coefficients of gene *i*. Then, we performed zero-mean normalization on the ADPS of the patients and categorized them into high ADPS and low ADPS groups.

### Development of potential therapeutic agents

We followed the protocol outlined by Yang et al. [39] to identify potential agents for LUAD patients with high ADPS: (1) We downloaded drug sensitivity data for cancer cell lines (CCLs) from the Cancer Therapeutic Response Portal (CTRP, includes 481 compounds over 835 CCLs, https://portals.broadinstitute.org/ctrp) and profiling relative inhibition simultaneously in mixtures (PRISM, includes 1448 compounds over 482 CCLs, https://www.theprismlab.org/) datasets, as well as expression data of CCLs from the Cancer Cell Line Encyclopedia (CCLE, https://portals.broadinstitute.org/ccle/) database. (2) The CTRP and PRISM datasets possess AUC values, and decreased AUC values indicate heightened responsiveness to this compound. (3) Using the Wilcoxon rank-sum test, we conducted a differential analysis of drug response between the top 10% (high ADPS group) and bottom 10% (low ADPS group) of samples. We set a threshold of log2FC >0.1 to identify compounds with significantly lower AUC values in the high ADPS group. (4) Furthermore, we employed Spearman correlation analysis to detect compounds with AUC values that showed negative correlation coefficients (using a threshold of R < -0.4) for subsequent screening. (5) We further identified potential agents by taking the intersection of the compounds obtained from steps (3) and (4). In the end, according to differential expression analysis, we identified potential agents using Connectivity Map (CMap, https://clue.io/) [40] to further verify the agents derived from the CTRP and PRISM databases.

### Multi-omics alteration characteristics targeting ADPS

GISTIC 2.0 analysis (https://gatk.broadinstitute.org) was applied to distinguish recurrently amplified and deleted regions for genomic alterations. TCGA GDC data Portal was used to download “maf” data for LUAD samples (VarScan2Variant Aggregation and Masking; https://portal.gdc.cancer.gov). The TMB score with high ADPS and low ADPS groups was further calculated according to the “maftools” package [41]. The fraction of genome alteration (FGA), the fraction of genome gained (FGG), as well as the fraction of genome lost (FGL), were calculated as follows: FGA = total CNV/all bases, FGG = gain bases/all bases, and FGL = loss bases/all bases. These metrics were used to evaluate the extent of genomic alterations in the samples.

### Statistical analysis

A Wilcoxon rank-sum test was utilized to compare the continuous variables. Cox regression was performed using the “survival” package to analyze the relationship between variables and survival outcomes. The glm function was utilized for logistic regression to examine the relationship between variables and binary outcomes. The log-rank *P* test was utilized for the Kaplan-Meier analysis. The ROC curve was plotted via the “timeROC” package and the calibration curve was plotted using the “rms” package. Statistical significance was set at *P* < 0.05.

### Availability of data and materials

The original contributions presented in the study are included in the Article/Supplementary Materials; further inquiries can be directed to the corresponding authors.

## RESULTS

### Heterogeneity of aggrephagy genes across cells

**Figure 1:**
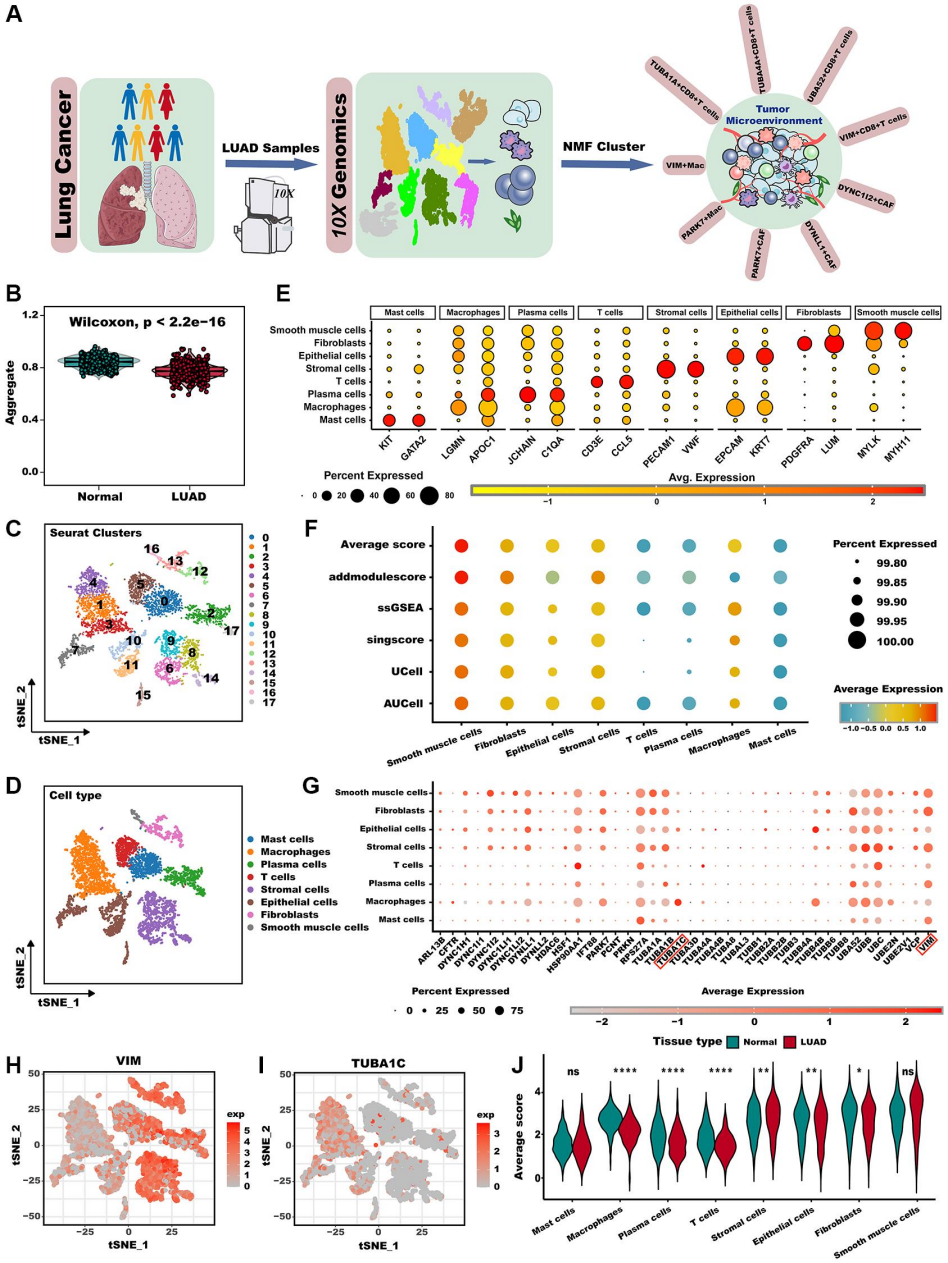
**Overview of aggrephagy gene expression in the scRNA-seq for LUAD.** (**A**) The overall design of this study. (**B**) The GSVA scores based on the aggrephagy gene set were compared between normal and LUAD samples. (**C**) t-SNE plot colored by 18 cluster of cells. (**D**) Cell type annotations clustered by Seurat t-SNE in the scRNA-seq data. (**E**) Dot plot showing representative marker genes for each cell type. (**F**) Bubble plot showing aggrephagy scores for each cell type. (**G**) Dot plot showing the expression level of aggrephagy genes in all cell types. (**H**, **I**) Expression of example genes VIM (**H**), and TUBA1C (**I**) in different cell types. (**J**) The difference in average aggrephagy score in each cell type in normal and LUAD samples. ^*^p < 0.05; ^**^p < 0.01; ^***^p < 0.001. Abbreviation: ns: not significant.

The workflow chart illustrates the general design of this study (Figure 1A). We found that the aggrephagy score was significantly increased in normal samples compared LUAD samples analyzed via the ssGSEA algorithm (Figure 1B, *P* < 0.001). We then examined the landscape of aggrephagy on each cell type by using the scRNA-seq data of LUAD. After using the Seurat pipeline, a total of 12,554 cells were divided into 18 clusters (Figure 1C) and were annotated with major cell types based on classical marker genes, including mast cells, macrophages, plasma cells, T cells, stromal cells, epithelial cells, fibroblasts, and smooth muscle cells (Figure 1D). Marker genes for each cell population showed a clear separation between each cell type (Figure 1E). To further investigate whether aggrephagy activity was dynamic among TME in LUAD scRNA-seq level, we quantified aggrephagy score using ssGSEA, AUCell, Ucell, addmodulescore, and singsore algorithms (Figure 1F). Interestingly, the outcomes demonstrated relatively heightened aggrephagy score in smooth muscle cells, fibroblasts, epithelial, stromal, and macrophage, while lower in T, plasma, and mast cells. Moreover, a comparison between average aggrephagy score in LUAD and normal tissues unveiled intriguing observations: macrophage, plasma, T, epithelial, and fibroblasts within tumors exhibited notably low average aggrephagy score, while stromal cells exhibited notably high average aggrephagy score (Figure 1J). As a result of comparing the expression patterns of aggrephagy genes across cell types in LUAD, we found that aggrephagy genes also showed high heterogeneity among cell types (Figure 1G). For instance, VIM exhibited higher expression levels in fibroblasts, and plasma cells, while showed lower expression levels in T cells and macrophages (Figure 1H). TUBA1C is highly expressed in macrophages and epithelial cells, but shared lower expression levels in other cells (Figure 1I), indicating the necessity for further investigation of aggrephagy genes by targeting specific cell types.

### Novel aggrephagy-mediated fibroblasts contribute to the TME of LUAD

**Figure 2:**
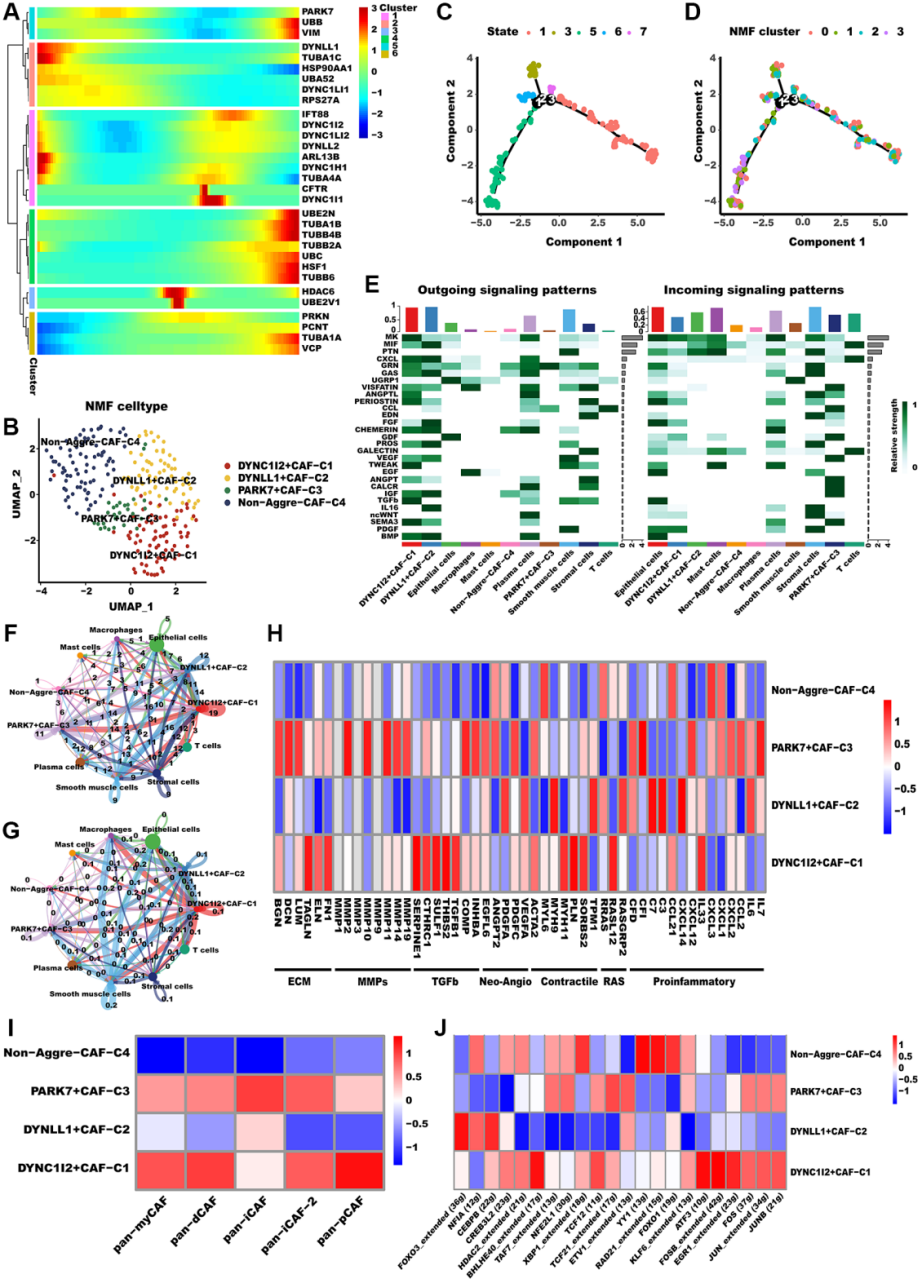
**Aggrephagy genes modified the features of CAF.** (**A**) Pseudotime trajectory analysis of aggrephagy genes in CAFs. (**B**) NMF clustering and annotation in CAFs classified by aggrephagy gene expression features. (**C**, **D**) The developing status of NMF-based CAF clusters obtained in pseudotime analysis. (**E**) A heat map summarizing the outgoing (secreting) and incoming (target) signal pathways of NMF-based aggrephagy-related CAF subtypes and other cell types. (**F**, **G**) The number (**F**, number of interactions) and weight (**G**, interaction weights/strength) of cell-cell interactions between Agg-related CAF subtypes and other cell types. (**H**) Heatmap showing the different average expression of common signaling pathway genes in the aggrephagy-related CAF subtypes, including collagens, ECM, MMPs, TGFb, Neo-Angio, Contractile, RAS and Proinflammatory. (**I**) Different aggrephagy-related CAF subtypes were correlated with the previous signatures. (**J**) Heatmap showing the significantly different activities of TFs among aggrephagy-related CAF subtypes by comparing the average AUC using pySCENIC in Python.

Recent research has recognized CAFs as a key factor in TME, and they are emerging as a research hotspot [42]. Advancements in single-cell analysis techniques have led to a more comprehensive comprehension of the diversity and role of CAFs. For instance, according to one of the most well-established categorizations of CAFs, CAFs can be categorized as myCAF (myfibroblastic CAF), dCAF (development CAF), iCAF (inflammatory CAF), as well as pCAF (Pdpn CAF). Distinctive characteristics differentiate various subgroups of CAFs. Specifically, myCAF subset is situated in proximity to cancer cell nests and is characterized by elevated expression levels of both FAP+ and ꬰ-SMA. Conversely, the iCAF subset is localized far from cancerous cells and is marked by low expression levels of ꬰ-SMA but high expression levels of IL-6; the dCAF subset is distinguished by the activation of genes associated with diverse types of stem cells [43]. Similarly, to determine whether aggrephagy expression has an effect on CAFs, we performed a dimension reduction analysis. Pseudotime analysis reveals that aggrephagy genes are expressed at various developmental stages (Figure 2A). For instance, the early development stages of CAFs were characterized by the significant expression of TUBA4A, TUBA1C, and ARL13B, whereas TUBB4B, VIM, and TUBA1B were the feature of late development. Based on the NMF algorithm, CAFs were further divided into four clusters, and we identified that DYNC1I2+CAF−C1, DYNLL1+CAF−C2, PARK7+CAF−C3, as well as Non−Aggrephagy−CAF−C4 subtypes (Figure 2B, Supplementary Table 2). The developing status of NMF-based CAF clusters varied greatly as shown in pseudotime analysis (Figure 2C, 2D). Interestingly, using the Cellchat analysis, DYNC1I2+CAF−C1, DYNLL1+CAF−C2, and PARK7+CAF−C3 presented more and tighter connections with other cell types than Non-Aggre-CAF-C4 (Figure 2E–2G). GSVA was employed to derive scores for established classical CAF phenotype markers, with the aim of uncovering the possible phenotypes and functions of the aggrephagy CAF subtypes [44, 45] (Figure 2I). DYNC1I2+CAF−C1 showed the most prominent scores of pan-myCAF, pan-dCAF, and pan-pCAF, while PARK7+CAF−C3 exhibited the prominent scores of pan-iCAF. In contrast, Non-Aggrephagy-CAF-C4 had the lowest score among all classical CAF subtypes. In addition, we also investigated important CAF phenotype markers, like pro-inflammatory genes, neo-angiogenic genes, and MMPs. From the pathway heatmap (Figure 2H), aggrephagy-CAF subtypes had a significantly different expression of common pathway genes. PARK7+CAF−C3 had significantly higher expression of MMPs, ECM, Neo-Angio, and proinflammatory pathways genes, DYNC1I2+CAF−C1 had significantly higher expression of TGFb and RAS pathways gens, while Non-Aggre-CAF-C4 had lower expression of above pathways genes. Ultimately, we compared TF regulation features and subtype-specific TFs between aggrephagy-related CAFs as well as non-aggrephagy-related CAFs. We found that the average activities of TFs including FOS and ATF3 were exclusively higher in DYNC1I2+CAF−C1, while downregulated in DYNLL1+CAF−C2 except for FOXO3, NFIA, and CEBPB (Figure 2J). In aggregate, these discoveries imply that aggrephagy could exert a significant influence on CAF remodeling within the TME of LUAD.

### Aggrephagy participate in TAMs metabolism and polarization

**Figure 3:**
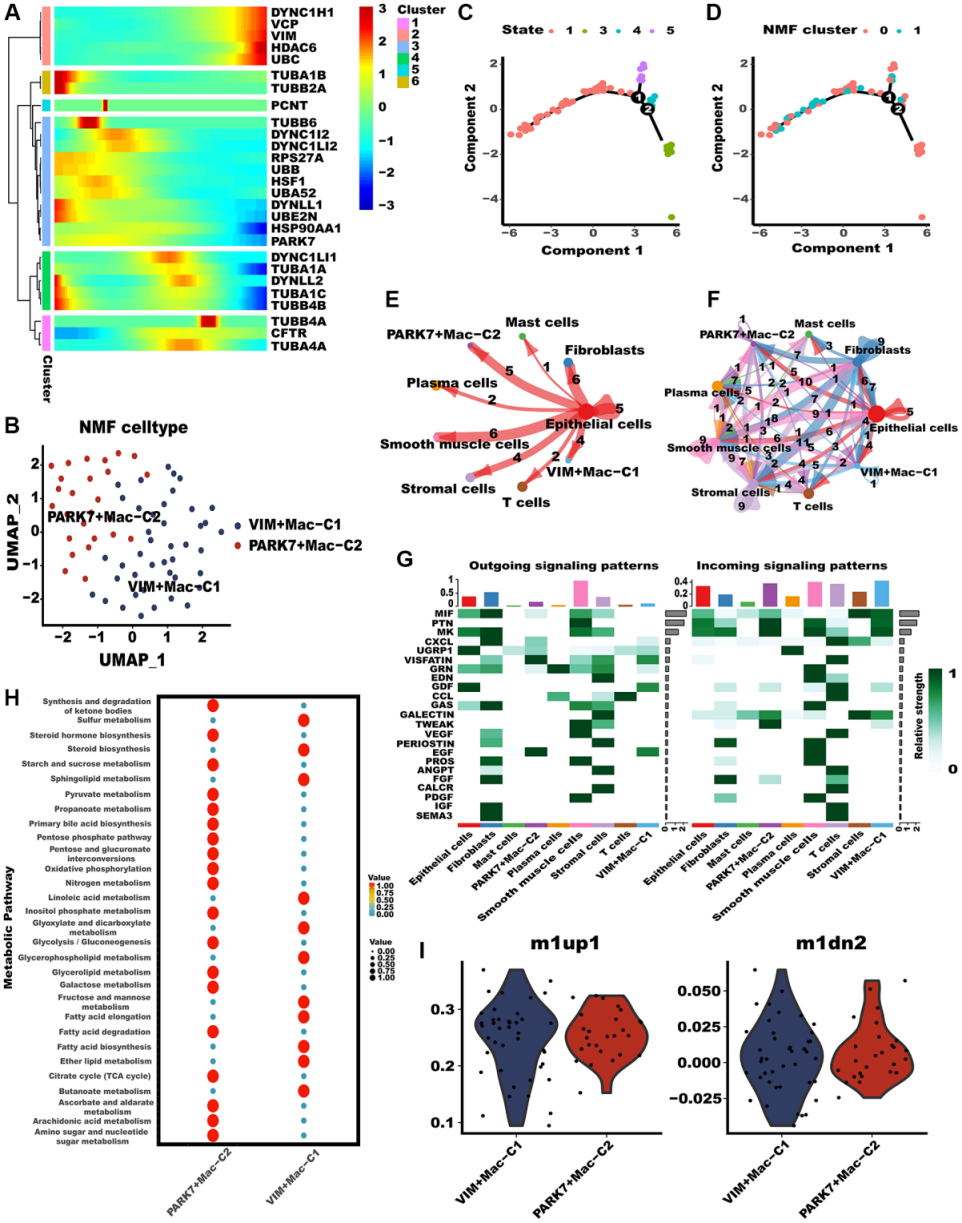
**Tumor-associated macrophages (TAMs) differed in metabolism and polarization during aggrephagy.** (**A**) Pseudotime trajectory analysis of aggrephagy genes in TAMs. (**B**) NMF clustering and annotation in TAMs classified by aggrephagy gene expression features. (**C**, **D**) The developing status of NMF-based TAM subtypes obtained in pseudotime analysis. (**E**, **F**) The number and weight of cell-cell interactions between aggrephagy-related TAM subtypes and other cell types. (**G**) A heat map summarizing the outgoing (secreting) and incoming (target) signal pathways of NMF-based aggrephagy-related TAM subtypes and other cell types. (**H**) Metabolic status of aggrephagy-related TAM subtypes analyzed by scMetabolism. (**I**) M1-like/M2-like phenotype scoring among different aggrephagy-related TAM subtypes.

Subsequently, we investigated the potential impact of aggrephagy on the phenotypes and functions of TAMs. Throughout the pseudotime analysis, aggrephagy genes were observed to exhibit diverse expression patterns at different stages of macrophage development, implying their complex and dynamic roles in regulating the differentiation and function of macrophages (Figure 3A). NMF with aggrephagy genes separated macrophages into VIM+Mac−C1 and PARK7+Mac−C2 (Figure 3B, Supplementary Table 3). The developing status of NMF-based TAM clusters varied greatly as shown in pseudotime analysis (Figure 3C, 3D). Further, VIM+Mac−C1 and PARK7+Mac−C2 had comparatively stronger interactions with other cellular components, especially with epithelial cells (Figure 3E, 3F). We inferred the specific pathways of intercellular communication and found that mainly outgoing (secreting) of VIM+Mac−C1 and PARK7+Mac−C2 were EGF and VISFATIN, and incoming (target) signal pathways were PTN and MK (Figure 3G). A tumor microenvironment induces metabolic reprogramming of macrophages, leading to protumor macrophages having an overactive metabolism [37], we conducted scMetabolism algorithm to explore the metabolic heterogeneity in different aggrephay TAM subtypes (Figure 3H). Interestingly, the result revealed that the two TAM subtypes exhibit unique metabolic activation pathways, indicating that aggrephagy could be a critical modulator of metabolic regulation. For instance, PARK7+Mac−C2 was distinguished by the upregulation of multiple metabolic pathways such as glycolysis, TCA cycle, fatty acid degradation, as well as oxidative phosphorylation, while VIM+Mac−C1 was distinguished by the upregulation of fatty acid elongation and ether lipid metabolism. In addition, we computed the M1-like/M2-like polarization scores of different aggrephagy TAMs subtypes [46]. Notably, PARK7+Mac−C2 and VIM+Mac−C1 exhibited a preference for expressing genes that were upregulated in M1 macrophages, suggesting their enhanced M1-like anti-tumor functions (Figure 3I).

### Aggrephagy contributed to the subgroup formation in CD8+T cells

**Figure 4:**
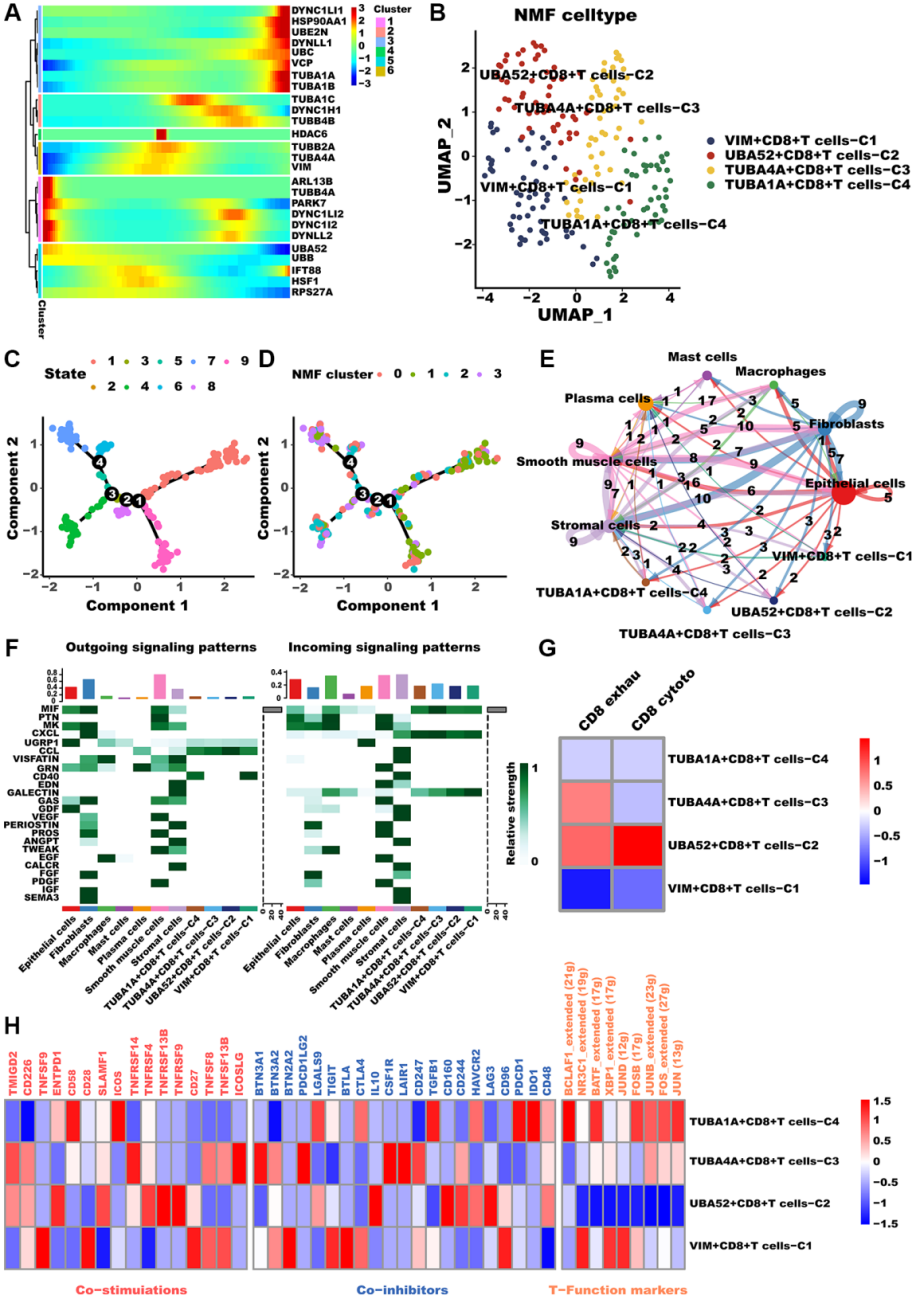
**CD8+T cells differed in metabolism and polarization during aggrephagy.** (**A**) Pseudotime trajectory analysis of aggrephagy genes in CD8+T cells. (**B**) NMF clustering and annotation in CD8+T cells classified by aggrephagy gene expression features. (**C**, **D**) The developing status of NMF-based CD8+T cells subtypes. (**E**) The number and weight of cell-cell interactions between aggrephagy-related CD8+T cells subtypes and other cell types. (**F**) A heat map summarizing the outgoing (secreting) and incoming (target) signal pathways of NMF-based aggrephagy-related CD8+T cells subtypes and other cell types. (**G**) Heatmap showing the comparison of CD8+T cell function signatures (exhaustion score and T cytotoxic score) between aggrephagy-related CD8+T cells subtypes. (**H**) Heatmap showing significantly different features among aggrephagy-related CD8+T cells subtypes, including Co-stimulations (left), Co-inhibitors (middle), and TFs (right).

As CD8+T cells are known to significantly impact tumor metastasis and treatment outcomes, we conducted a comprehensive analysis of these cells [47]. Likewise, Monocle2 revealed that aggrephagy genes occupy different developmental stages (Figure 4A). TUBA1A, concordant with CAFs, was highly expressed during late development, while PARK7 and UBA52 were expressed during early development. Furthermore, CD8+T cells were categorized as four clusters via the NMF algorithm, and the developing status of NMF-based CD8+T cells varied greatly (Figure 4C, 4D, Supplementary Table 4), as well as named them as VIM+CD8+T cells C1, UBA52+CD8+T cells C2, TUBA4A+CD8+T cells C3, and TUBA1A+CD8+T cells C4, respectively (Figure 4B). Similarly, the aforementioned aggrephagy CD8+ T cells subtypes were found to have more extensive and robust interactions with other components of the TME, indicating their potential role in orchestrating the tumor immune microenvironment (Figure 4E). Furthermore, we inferred the specific pathways of intercellular communication and found that VIM+CD8+T cells C1 and TUBA1A+CD8+T cells C4 presented stronger activity in the CD40 pathways compared to UBA52+CD8+T cells C2, TUBA4A+CD8+T cells C3 (Figure 4F). We computed the T exhaustion and T cytotoxic scores for these aggrephagy related CD8+ T cell subtypes, utilizing a previously published panel for calculating scores that evaluate overall functions and phenotypes. These scores play a crucial role in determining the effects of ICB and its significance in LUAD progression [48]. Interestingly, we found that UBA52+CD8+T cells C2 showed higher T cytotoxic scores, TUBA4A+CD8+T cells C3 demonstrated higher T exhaustion scores but lower T cytotoxic scores, while VIM+CD8+T cells C1 and TUBA1A+CD8+T cells C4 presented lower both scores (Figure 4G), indicating TUBA4A likely to be involved in CD8+T cell exhaustion, result in tumor immune escape. In addition, we performed a comparison of the mean expression levels of genes related to T cell function and immune checkpoint inhibitors across these subtypes of CD8+ T cells. Consequently, VIM+CD8+T cells C1, UBA52+CD8+T cells C2, TUBA4A+CD8+T cells C3, and TUBA1A+CD8+T cells C4 exhibited different immune function-related terms (Figure 4H). Ultimately, we investigated the regulatory mechanisms through network regulatory analysis. TUBA1A+CD8+T cells C4 demonstrated apparent activation of TFs including FOS, JUNB, FOSB, BCLAF1, and BATF (Figure 4H). In conclusion, our investigation has illustrated the role of aggrephagy in the restructuring of CD8+ T cells within the TME of LUAD.

### Aggrephagy-mediated TME remodeling contributes to prognosis and immunotherapy response in LUAD

**Figure 5:**
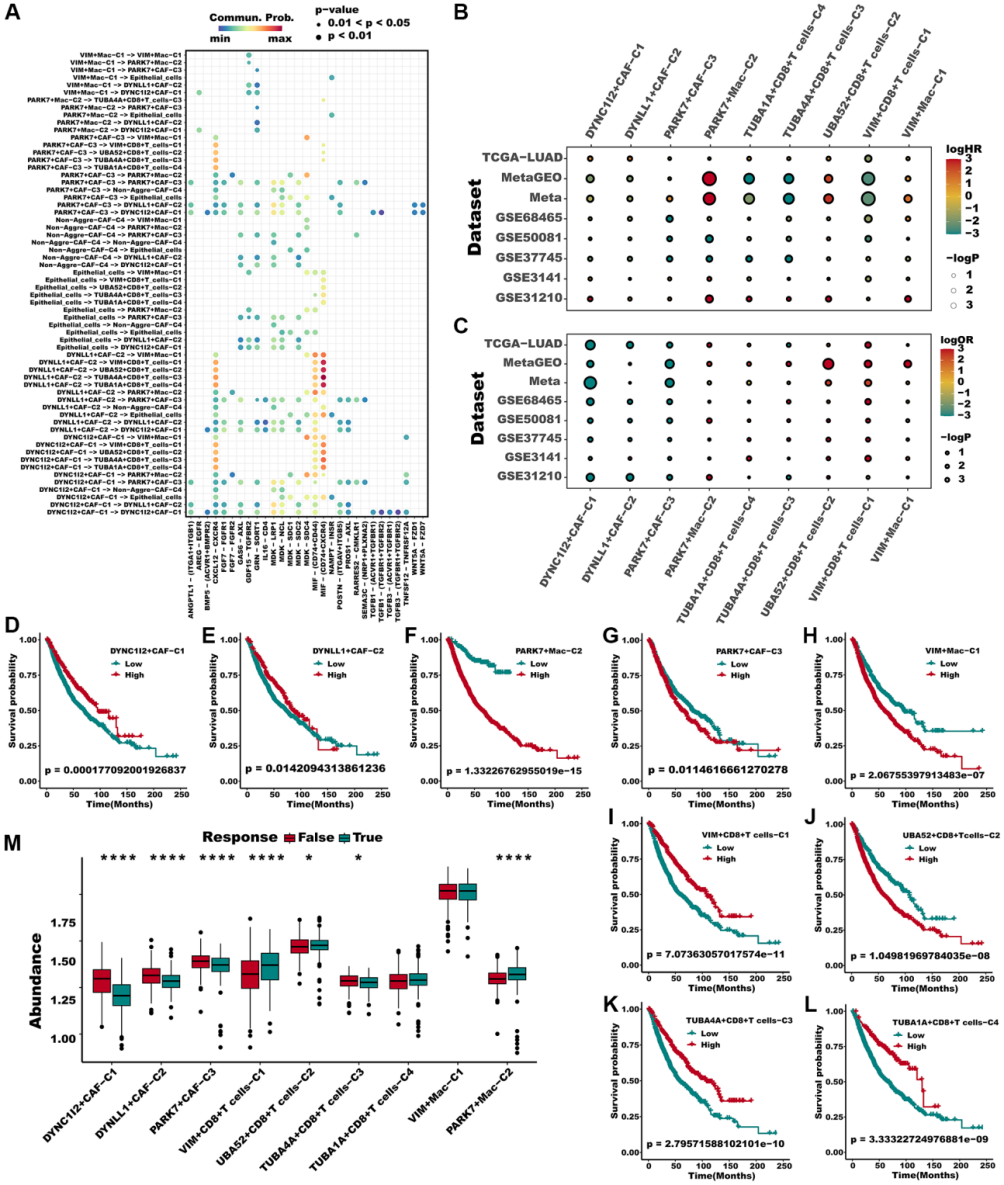
**Multiple aggrephagy cell subtypes influenced the prognosis and immunotherapy response of LUAD patients.** (**A**) The landscape of all cell-cell communication within all identified aggrephagy subtypes. (**B**) A hazard ration of different aggrephagy subtypes in 8 LUAD cohorts. (**C**) Odd ratio produced by logistic regression of different subtypes in 8 cohorts. (**D**–**L**) All aggrephagy cell subtypes could distinguish the survival of patients in Meta cohort. (**M**) Various aggrephagy-related subtypes demonstrated significantly different infiltration in responders and non-responders of ICB in TCGA cohorts, as predicted by TIDE. Mann Whitney-Wilcoxon test was applied between responders and non-responders. ^*^p < 0.05; ^**^p < 0.01; ^***^p < 0.001. Abbreviation: ns: not significant.

**Figure 6:**
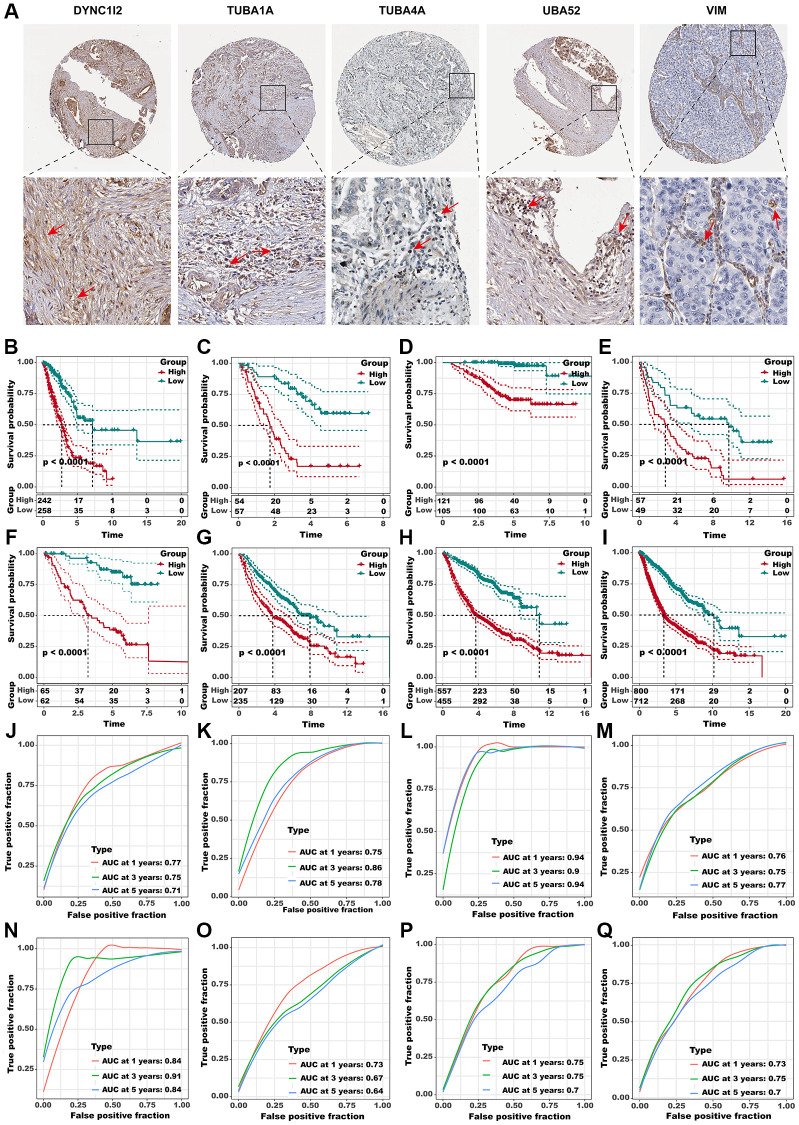
**Identification of the hub predictive genes to construct ADPS.** (**A**) IHC staining images of five crucial aggrephagy genes. Panel1: DYNC1I2+ fibroblasts were marked with a red arrow. Protein was mainly expressed on cell membrane (source HPA. Patient ID: 448, female, age 76 years). Panel2: TUBA1A+ T cells were marked with a red arrow. Protein was mainly expressed on cytoplasmic/membranous (source HPA. Patient ID: 448, female, age 76 years). Panel3: TUBA4A+ T cells were marked with a red arrow. Protein was mainly expressed on cytoplasmic/membranous (source HPA. Patient ID: 2403, female, age 65 years). Panel4: UBA52+ T cells were marked with red arrows. Protein was mainly expressed in the nucleus (source HPA. Patient ID: 2393, female, age 54 years). Panel5: VIM + Macrophages were marked with red arrows. Protein was mainly expressed in the cytoplasmic/membranous (source HPA. Patient ID: 1421, female, age 76 years). (**B**–**I**) Kaplan-Meier survival curves of the ADPS regarding OS in the TCGA (**B**), GSE3141 (**C**), GSE31210 (**D**), GSE37745 (**E**), GSE50081 (**F**), GSE68465 (**G**), MetaGEO (**H**), and Meta cohorts (**I**). (**J**–**Q**) Time-dependent ROC curves of the ADPS regarding 1-, 3-, and 5-year OS in the TCGA (**J**), GSE3141 (**K**), GSE31210 (**L**), GSE37745 (**M**), GSE50081 (**N**), GSE68465 (**O**), MetaGEO (**P**), and Meta cohorts (**Q**).

We have set out to investigate whether the newly defined aggrephagy subtypes have an impact on the survival rates of patients diagnosed with LUAD. Initially, we depicted the intercellular communication among all the aggrephagy-related subtypes, which elucidated all the possible connections between these subtypes (Figure 5A). Then we utilized ssGSEA algorithm to compute the enrichment score of each aggrephagy subtype based on the corresponding differentially expressed genes (Supplementary Table 5) and investigated their prognostic significance in LUAD patients across multiple cohorts. Through the implementation of univariate Cox regression analysis, we acquired the hazard ratio of each aggrephagy cell subtype in TCGA, GSE68465, GSE50081, GSE37745, GSE3141, GSE31210, Meta-GEO, and Meta cohorts, which showed that PARK7-Mac-C2, UBA52+CD8+T_cells−C2, PARK7+CAF-C3, and VIM+Mac-C1 were poor prognostic factors (Figure 5B, Supplementary Table 6). In addition, certain aggrephagy-related cell subtypes could distinguish the OS in TCGA, GSE68465, GSE50081, GSE37745, GSE3141, GSE31210, and Meta-GEO cohorts (Supplementary Figure 1A–1AJ). Remarkably, all cell subtypes related to aggrephagy were able to differentiate overall survival (OS) among meta-cohorts, which included 1512 patients with LUAD (Figure 5D–5L). Having established the potential role of aggrephagy in shaping the TME, we proceeded to investigate whether the aggrephagy-based patterns of TME had an impact on the response to ICB therapy. We employed the TIDE algorithm to predict the response of each patient to ICB therapy across multiple cohorts. Additionally, we observed that in the TCGA cohort, DYNC1I2+CAF−C1, DYNLL1+CAF−C2, and PARK7+CAF−C3, TUBA4A+CD8+T_cells−C3 were downregulated in responders, suggesting that these cells may be linked to ICB resistance (Figure 5M), which was verified in other cohorts (Supplementary Figure 2). Utilizing logistic regression, we obtained the odds ratio value for response prediction, unveiling the detrimental impact of these cells (Figure 5C, Supplementary Table 7). To verify the presence of the above cell types, we used IHC to observe cellular localization of DYNC1I2, TUBA1A, TUBA4A, UBA52 and VIM. We discovered that some of the fibroblasts in stromal did express DYNC1I2. (Figure 6A, first panel). Similarly, TUBA1A+ T cells were observed, showing significant positivity, from which immune cells were seen to exude (Figure 6A, second panel). TUBA4A was expressed on the cell nuclear of some T cells, with stromal cell nearby (Figure 6A, third panel). As a highly conserved nuclear and cytoplasmic protein, UBA52 was expressed in some T cells. As an immune cells, we also observed a high abundance of fibroblasts around UBA52+ T cells in pathological sections (Figure 6A, fourth panel). As we known, VIM was an epithelial-mesenchymal transition biomarker, highly expressed in stromal cell and tumor cell. In pathological sections, we found VIM was expressed in some macrophages, with tumor cell nearby. (Figure 6A, fifth panel).

### Aggrephagy-derived prognostic score (ADPS)

Subsequently, considering the concordant performances of aggrephagy subtypes in anticipating the overall survival outcomes as well as immunotherapy response in multiple LUAD cohorts, we retired marker genes and a total of 571 genes from aggrephagy-related subtypes were obtained (Supplementary Table 8). A total of 114 genes were found to have prognostic value through the assessment of each gene using univariate Cox regression analysis (Supplementary Figure 3A). To streamline the number of genes, we utilized LASSO Cox regression analysis, which resulted in 32 remaining genes at a lambda value of 0.0252 (Supplementary Figure 3B, 3C). The ADPS was ultimately generated by performing multivariate Cox regression analysis (Supplementary Figure 3D, Supplementary Table 9). We calculated the ADPS for each patient according to the expression as well as weighted regression coefficients of the ADPS-related genes, and then partitioned them to high ADPS and low ADPS groups after z-mean normalization. K-M survival analyses revealed that the mortality rate in the high ADPS group was significantly higher than the low ADPS group in the training cohort (TCGA-LUAD, *n* = 500, *P* < 0.005), and other seven validation cohorts GSE3141 (*n* = 111, *P* < 0.0001), GSE31210 (*n* = 226, *P* < 0.0001), GSE37745 (*n* = 106, *P* < 0.0001), GSE50081 (*n* = 126, *P* < 0.0001), GSE68465 (*n* = 442, *P* < 0.0001), MetaGEO (*n* = 1012, *P* < 0.0001), and Meta cohort (*n* = 1512, *P* < 0.0001) (Figure 6B–6I). Moreover, in the TCGA cohort, the ADPS exhibited excellent performance, with time-dependent AUCs of 0.77, 0.75, and 0.71 at 1, 3, and 5 years ((Figure 6J). Comparable results were also acquired across the validation cohorts GSE3141 (0.75/0.86/0.78), GSE31210 (0.94/0.90/0.94), GSE37745 (0.76/0.75/0.77), GSE50081 (0.84/0.91/0.84), GSE68465 (0.73/0.67/0.64), MetaGEO (0.75/0.75/0.70), as well as Meta cohort (0.73/0.75/0.7), respectively (Figure 6K–6Q).

### Predictive value of ADPS for immunotherapy

**Figure 7:**
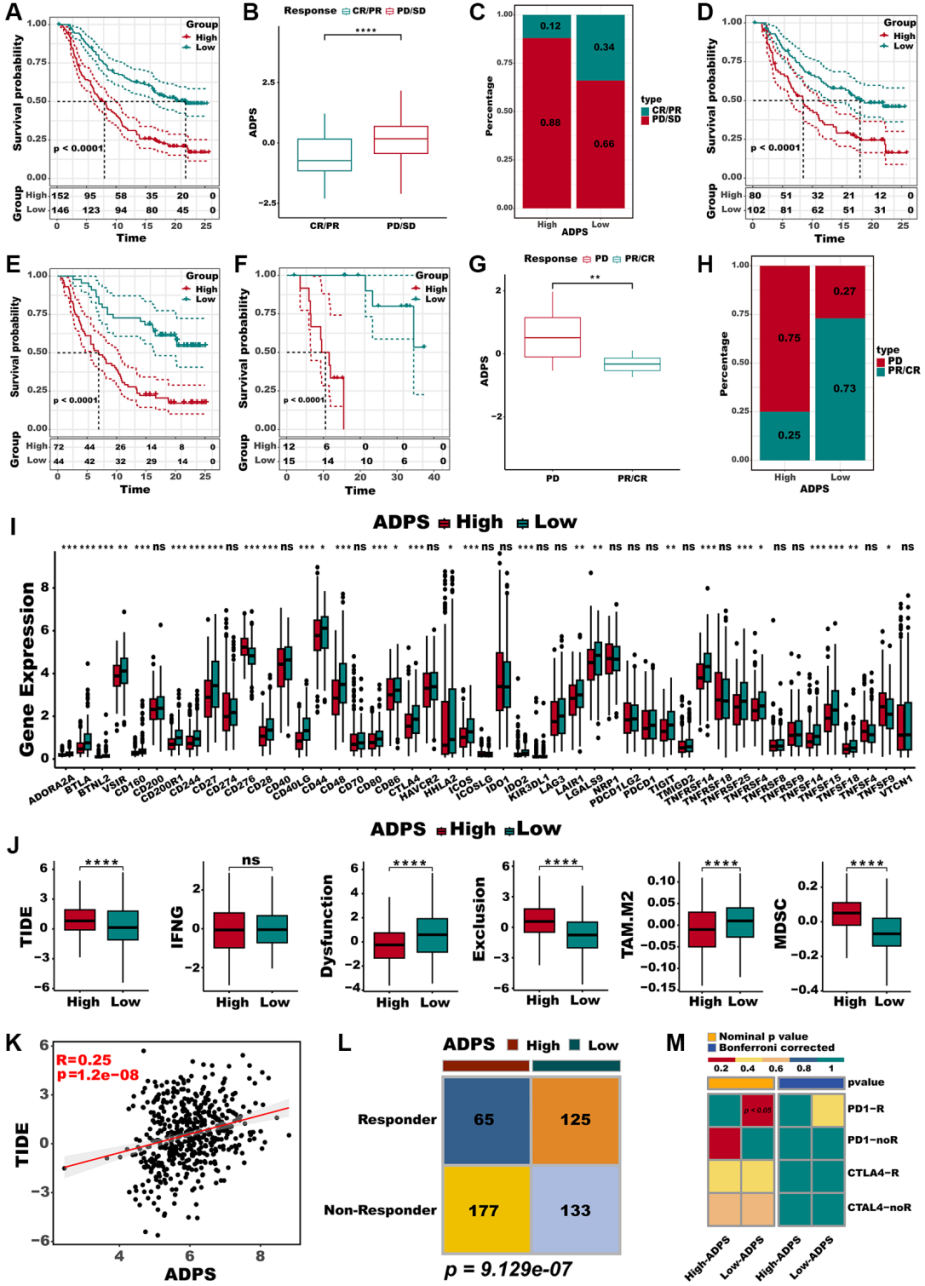
**Immunotherapy response prediction of the ADPS.** (**A**) Kaplan-Meier survival curves of the ADPS regarding OS in the IMvigor210 cohort. (**B**) Differences in ADPS among immunotherapy responses in the Imvigor210 cohort. (**C**) Distribution of immunotherapy responses among ADPS groups in the Imvigor210 cohort. (**D**) Prognostic differences between ADPS groups in early stage (stage I+II) patients in the Imvigor210 cohort. (**E**) Prognostic differences between ADPS groups in advanced stage (stage III+IV) patients in the Imvigor210 cohort. (**F**) Kaplan-Meier survival curves of the ADPS regarding OS in the GSE78220 cohort. (**G**) Differences in ADPS among immunotherapy responses in the GSE78220 cohort. (**H**) Distribution of immunotherapy responses among ADPS groups in the GSE78220 cohort. (**I**) Boxplot of relative expression levels at immune checkpoints between the high and low ADPS patients. (**J**) Boxplot of TIDE score between the high and low ADPS patients. (**K**) The relationship between the TIDE score and ADPS. (**L**) Contingency table between immunotherapy responses and ADPS groups based on TIDE algorithm. (**M**) Submap analysis of the two groups and 47 pretreated patients with comprehensive immunotherapy annotations. In submap analysis, a smaller *p*-value indicated a greater similarity of paired expression profiles. ^*^p < 0.05; ^**^p < 0.01; ^***^p < 0.001. Abbreviation: ns: not significant.

In addition, we evaluated the prognostic significance of ADPS about immunotherapy, utilizing the real-world cohort including IMvigor210 and GSE78220 datasets. Within the IMvigor210 cohort, which consisted of 298 patients, responses to anti-PD-L1 receptor blockers ranged from the complete response (CR) and partial response (PR) to stable disease (SD) and progressive disease (PD). A significant clinical benefit was observed in the low ADPS group of the IMvigor210 cohort compared with the high ADPS group (Figure 7A). CR/PR patients presented lower ADPS than SD/PD patients (Figure 7B, *P* < 0.0001). The low ADPS group displayed a higher percentage of CR/PR compared to the high ADPS group (Figure 7C). Survival differences between different ADPS groups were significant specifically in patients with Stage I+II (Figure 7D, *P* < 0.001), and in Stage III+IV patients (Figure 7E, *P* < 0.001). Our finding also indicated that patients with a low ADPS had a significantly better overall survival outcome compared to those with a high ADPS in the GSE78220 cohort (Figure 7F, *P* < 0.001). Besides, CR/PR patients also presented lower ADPS than SD/PD patients (Figure 7G, *P* < 0.01). Additionally, a higher percentage of SD/PD was observed in the high ADPS group as compared to the low ADPS group (Figure 7H). In addition to the abundance of immune checkpoints, we also noted a significantly higher expression of these checkpoints in the low ADPS group as compared to the high ADPS group (Figure 7I). Utilizing the TIDE web tool, we discovered that the low ADPS group had notably lower TIDE scores and higher rates of immunotherapy response compared to the high ADPS group (Figure 7J). There was a significant positive correlation observed between ADPS and TIDE score (Figure 7K). Responders to ICIs were found to be more prevalent among patients belonging to the high ADPS group, as predicted by the TIDE algorithm (Figure 7L, *P* < 0.001). Parallel to these findings, the subclass mapping analysis (Submap, modules in GenePattern, https://cloud.genepattern.org) also revealed similarities in expression patterns between patients with low ADPS and those with melanoma who responded to immunotherapy [49, 50] (A melanoma dataset that responded to immunotherapy was selected as the reference, while default settings were applied) responding to ICB (Figure 7M).

### Immune landscape for ADPS

**Figure 8:**
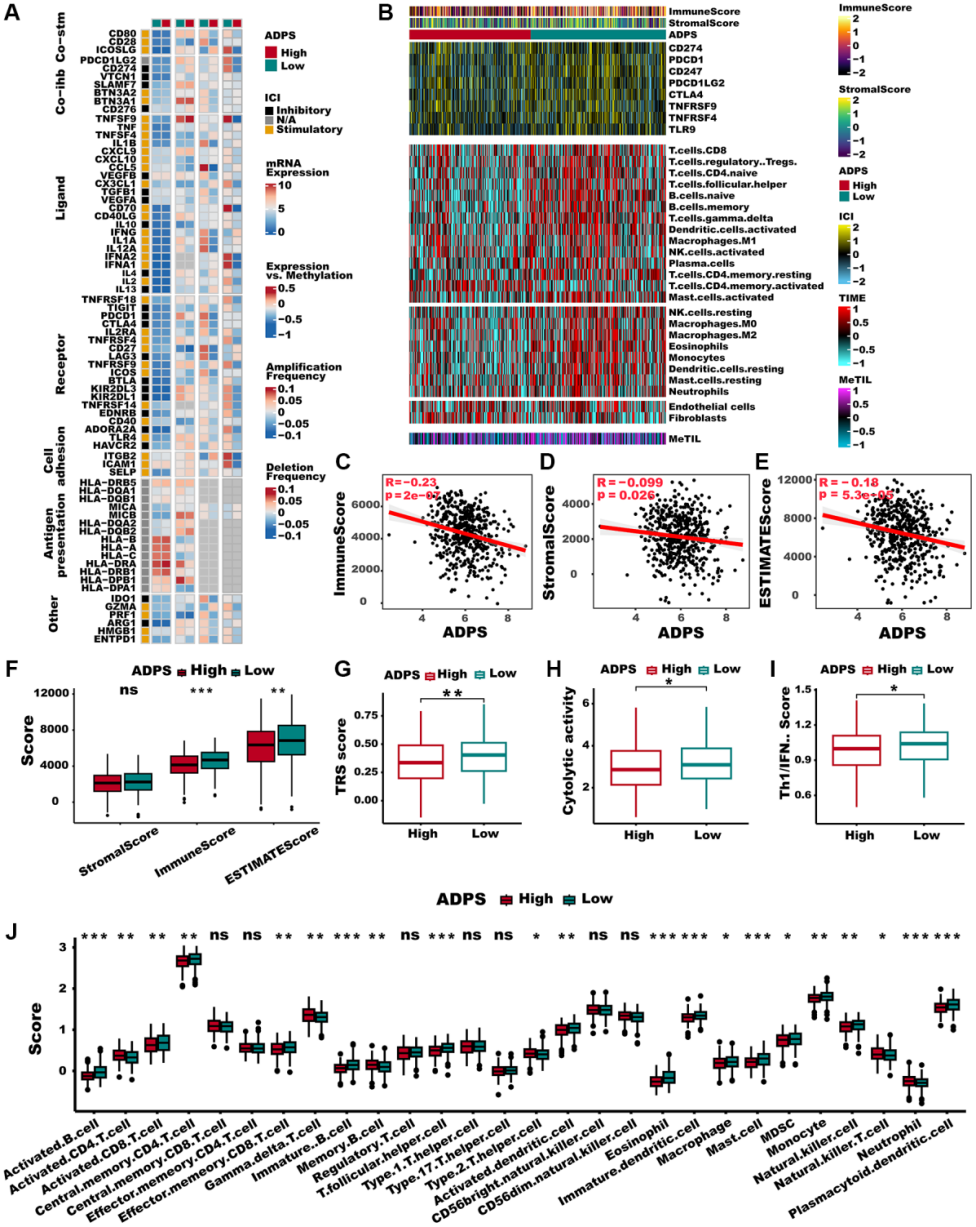
**Immune characteristics of the ADPS in the TCGA dataset.** (**A**) The correlation between the ADPS and immune modulators. (**B**) Heatmap exhibiting the immune score, stromal score, critical checkpoints, and cell types calculated through CIBERSORT analysis of the high and low ADPS groups. (**C**–**E**) Correlations between ADPS and immune score (**C**), stromal score (**D**), and ESTIMATE score (**E**). (**F**) Boxplot of relative stromal, immune, and ESTIMATE score between high and low ADPS groups. (**G**) Boxplot of relative TRS score between high and low ADPS groups. (**H**) Boxplot of relative cytolytic activity between high and low ADPS groups. (**I**) Boxplot of relative Th1/IFN score between high and low ADPS groups. (**J**) Boxplot of relative infiltrate abundance of 28 immune cell types between high and low ADPS groups. ^*^p < 0.05; ^**^p < 0.01; ^***^p < 0.001. Abbreviation: ns: not significant.

**Figure 9:**
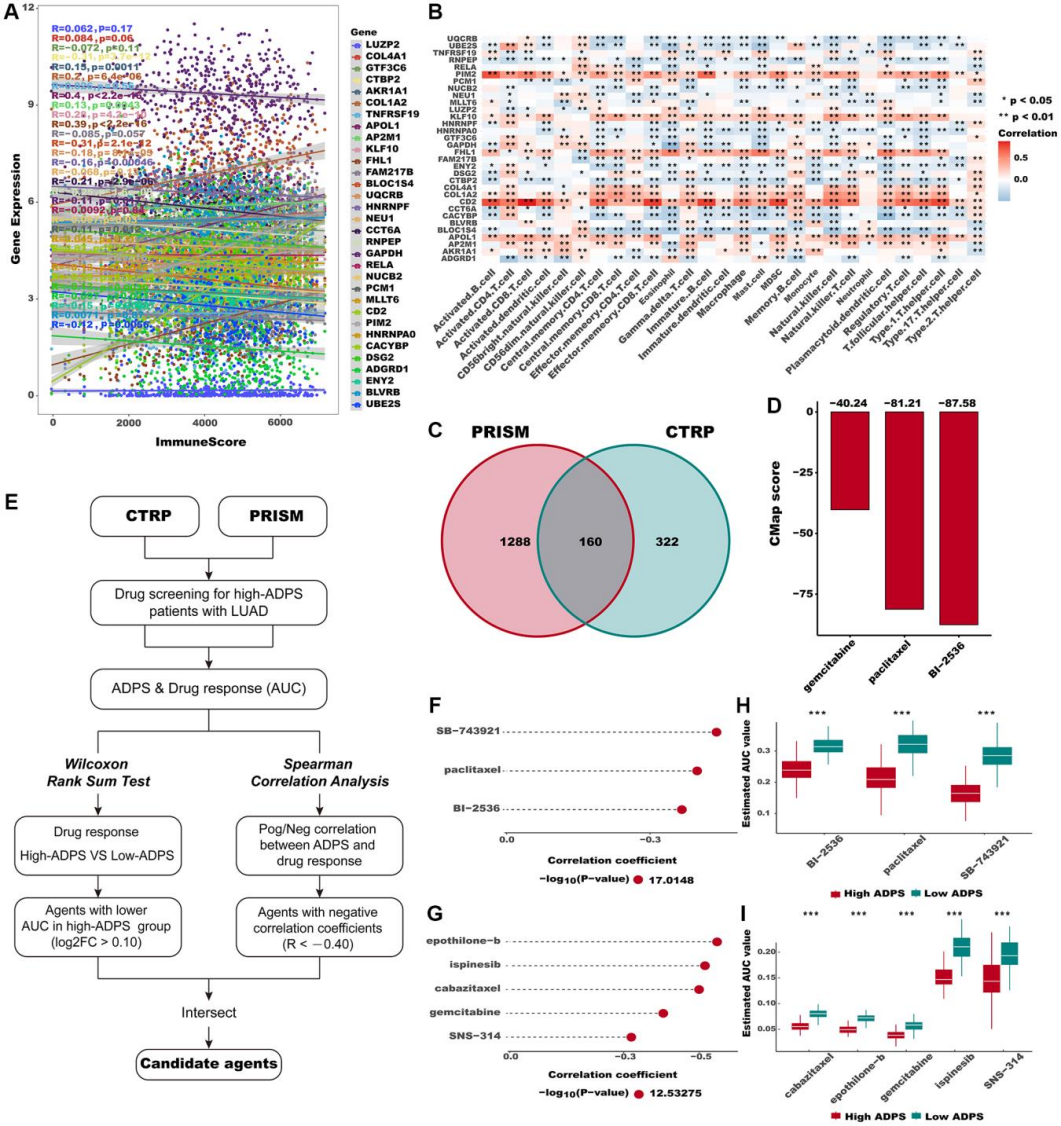
**Identification of candidate agents with higher drug sensitivity in high ADPS group.** (**A**) The correlation of gene expression and immune score. (**B**) The correlation of gene expression and immune cell types. (**C**) A Venn diagram for summarizing included compounds from CTRP and PRISM datasets. (**D**) Barplot of gemcitabine, paclitaxel, and BI-2536 CMap scores in patients with high ADPS. (**E**) Schematic outlining the strategy to identify agents with higher drug sensitivity in high ADPS patients. (**F**, **G**) The result of Spearman’s correlation analysis and differential drug response analysis of CTRP-derived compounds (**F**) and profiling relative inhibition simultaneously in mixtures (PRISM)-derived compounds (**G**). (**H**, **I**) The results of differential drug response analysis of CTRP -derived compounds (**H**) and PRISM -derived compounds (**I**), The lower the value of the y-axis, the greater the drug sensitivity. ^*^p < 0.05; ^**^p < 0.01; ^***^p < 0.001. Abbreviation: ns: not significant.

To explore the immune characteristics reflected by the ADPS, we investigated the association between the ADPS and immune cell type, immune scores, stromal scores, as well as critical immune checkpoint scores. Figure 8A, 8B demonstrated that the group with low ADPS exhibited elevated levels of immune infiltrating cells as well as immune modulators. This suggests the presence of an inflamed yet relatively immune-supportive microenvironment, which may be more conducive to benefiting from immunotherapy [51]. The immune score, stromal score, and ESTIMATE score had a negative correlation to ADPS, respectively (Figure 8C–8E). In parallel, patients in the low ADPS group had higher stromal, immune, and ESTIMATE scores compared to the high ADPS group (Figure 8F). Besides, we compared the status of the TRS score, Cytolytic activity, and Th1/IFN Score, which was more related to a more immunoreactive microenvironment between the two ADPS score groups [52, 53]. The results revealed that all of these indicators were significantly upregulated in the low ADPS group (Figure 8G–8I). According to 28 immune cells infiltration [54] assessed by ssGSEA (Figure 8J) and CIBERSORT algorithm (Supplementary Figure 4A), we further confirmed that the low ADPS group had markedly higher overall infiltration abundance than the high ADPS group. Thus, we defined the high ADPS group as “immune-cold” tumors and the low ADPS group as “immune-hot” tumors. Considering the upregulation of immune-related characteristics observed within the group of patients with a low ADPS, we sought to delve deeper into the biological mechanisms responsible for this phenomenon. KEGG-based GSVA analysis indicated the ADPS had a strong positive correlation with many pathways facilitating tumor growth, such as the P53 signaling pathway, cell cycle, glycolysis, and WNT signaling, while the immunological pathways, such as B/T cell receptor signaling pathways, cytokine receptor interaction pathway showed a weaker correlation with ADPS (Supplementary Figure 4B). Additionally, we further explored the immune characteristics from gene level, as displayed in Figure 9A, 9B, most ADPS-related genes were positively correlated with immune score as well as immune cell types.

### Searching for potential therapeutic agents for the high ADPS patients

The CTRP and PRISM databases comprise gene expression as well as drug sensitivity profiles from hundreds of cancer cell lines (CCLs), providing an opportunity to construct a model for predicting drug response. After removing duplicates, a total of 1770 unique compounds were obtained in the two datasets, with 160 compounds being shared between them (Figure 9C). Figure 9E demonstrates our strategic approach to developing potential agents for high ADPS patients. Subsequently, we implemented this methodology to pinpoint promising agent candidates for the high ADPS patients and generated three CTRP-derived agents (BI-2536, paclitaxel, and SB-743921), as well as five PRISM-derived agents (cabazitaxel, epothilone−b, gemcitabine, ispinesib, and SNS−314). The AUC values of these agents, as estimated, showed a statistically significant negative correlation with ADPS scores and were significantly lower in the high ADPS group (Figure 9F–9I). Furthermore, we leveraged the differential expression profiles of LUAD patients as well as normal samples to identify potential candidate compounds using the Cmap tool. Specifically, we aimed to identify agents whose gene expression patterns were opposite to the LUAD-specific expression patterns, indicating potential efficacy in suppressing LUAD tumor growth (i.e., gene expression increased in tumor tissues but decreased by treatment of certain compounds). Following the cross-referencing of the results from CTRP and PRISM, we were left with a trio of prospective agents: BI-2536, a PLK inhibitor, paclitaxel, and gemcitabine (Figure 9D). Among them, BI-2536, with a CMap score of −87.58, exhibited high sensitivity in LUAD patients, implying its potential as a therapeutic agent for those with high ADPS.

### Multi-omics alteration characteristics targeting ADPS

**Figure 10:**
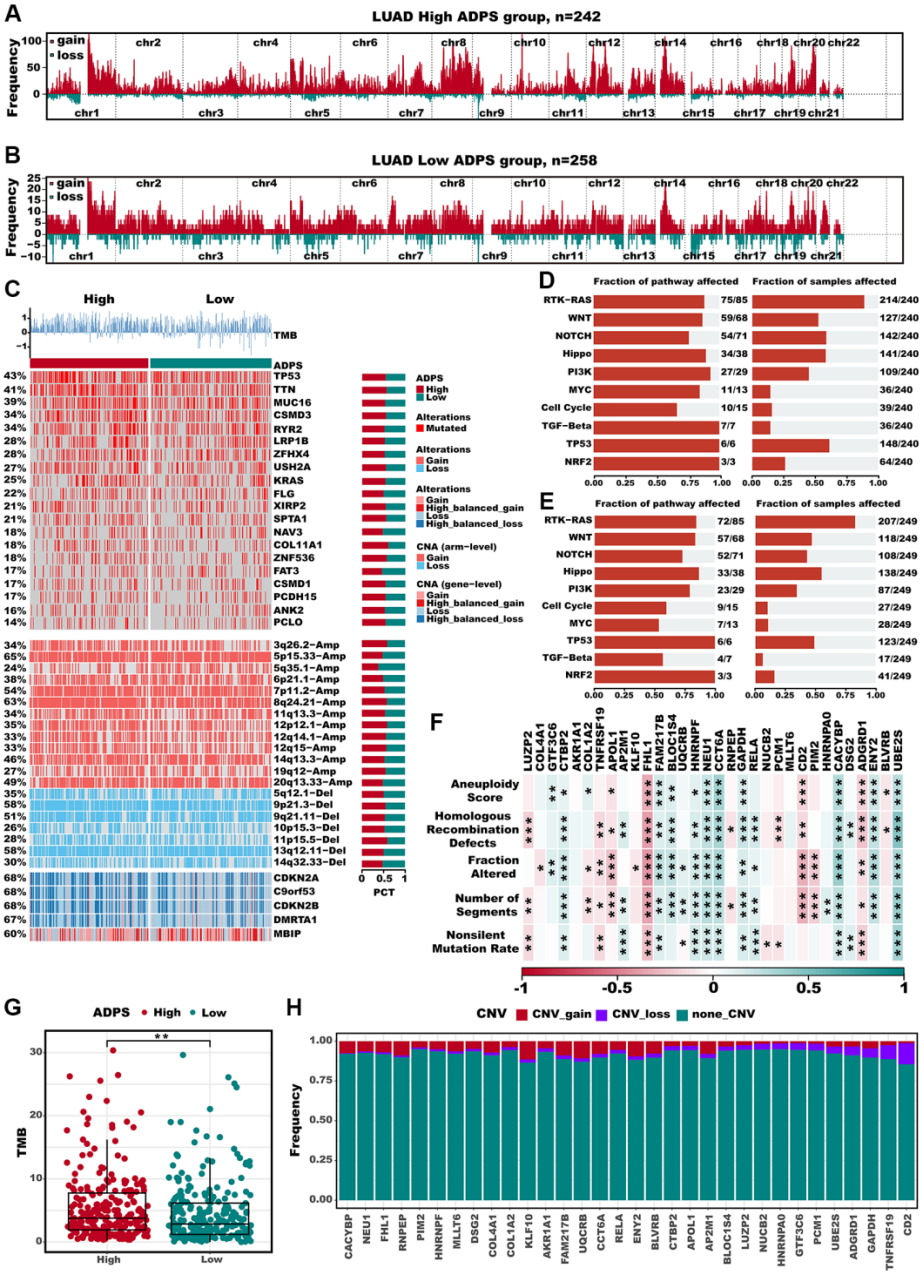
**Multi-omics alteration characteristics of the ADPS in the TCGA dataset.** (**A**, **B**) GISTIC 2.0-based chromosome amplifications and deletions in high (**A**) and low (**B**) ADPS groups. (**C**) Genomic alteration landscape according to ADPS. Tumor mutation burden (TMB), relative contribution of four mutational signatures, top 20 mutated genes and broad-level CNA (>20%). The proportion of the high and low ADPS groups in each alteration is presented in the right bar charts. (**D**, **E**) The mutation frequencies of nine common oncogenic pathways in the high ADPS (**D**) and low (**E**) groups. (**F**) Correlation heatmap of ADPS-related genes with Aneuploidy Score, Homologous Recombination Defects, Fraction Altered, Number of Segments, and Nonsilent Mutation Rate. (**G**) Difference of TMB score between high and low ADPS group. (**H**) CNV mutations (gain, loss, none) of ADPS-related genes. ^*^p < 0.05; ^**^p < 0.01; ^***^p < 0.001. Abbreviation: ns: not significant.

The differences in frequently altered chromosomes were detected in two ADPS groups (Figure 10A, 10B). We conducted an integrated analysis of mutations and copy number alterations (CNA, Figure 10C) to examine the genomic heterogeneity of the high and low ADPS groups. In the high ADPS group, we observed a higher frequency of mutations in classical tumor suppressor genes TP53 and CSMD3, as well as the oncogene KRAS, compared to the low ADPS group (Figure 10C). Additionally, compared to the low ADPS group, the high ADPS group showed significantly higher levels of amplification or deletion at the focal and chromosome arm levels, such as the amplification of 8q24.21, 12p12.1, as well as the deletion of 11p15.5, and 9p21.3 (Figure 10C). This finding was further supported at the gene level by the clear amplification of the oncogene MYC located at 8q24.21 and the distinct deletion of the tumor suppressor genes CDKN2A located at 9p21.3 (Figure 10C). Furthermore, we explored the mutation frequency of 10 major oncogenic pathways [55] between the high and low ADPS groups, and result found that both groups had detectable mutations in most of the oncogenic pathways, such as RTK-RAS, PI3K, TP53, NOTCH, and Hippo pathways (Figure 10D, 10E). We further explored the relationship between ADPS-related genes and LUAD by analyzing their correlations with different molecular signatures (Figure 10F). Our findings showed that FHL1 and ADGRD1 were strongly and positively correlated with molecular signatures associated with genomic instability in LUAD, including Aneuploidy Score, Homologous Recombination Defects, Fraction Altered, Number of Segments, as well as Nonsilent Mutation Rate. Additionally, the high ADPS group exhibited a higher TMB score in comparison to the low ADPS group (Figure 10G, *P* < 0.01). Only a small fraction of samples exhibited copy number variation (CNV) in the ADPS-related genes (Figure 10H). Additionally, we explored the single-nucleotide variant (SNV) mutations of the ADPS-related genes. It showed that COLA4A1, COL1A2, and LUZP2 had SNV mutations in more samples (Supplementary Figure 4C). Finally, we investigated the co-occurrence probability between ADPS-related genes and the top 10 genes with the highest mutation frequency. Supplementary Figure 4D reveals significant co-occurrence probabilities between CACYBP and ENU1, as well as between RELA and AP2M.

### Nomogram based on ADPS and clinical features

**Figure 11:**
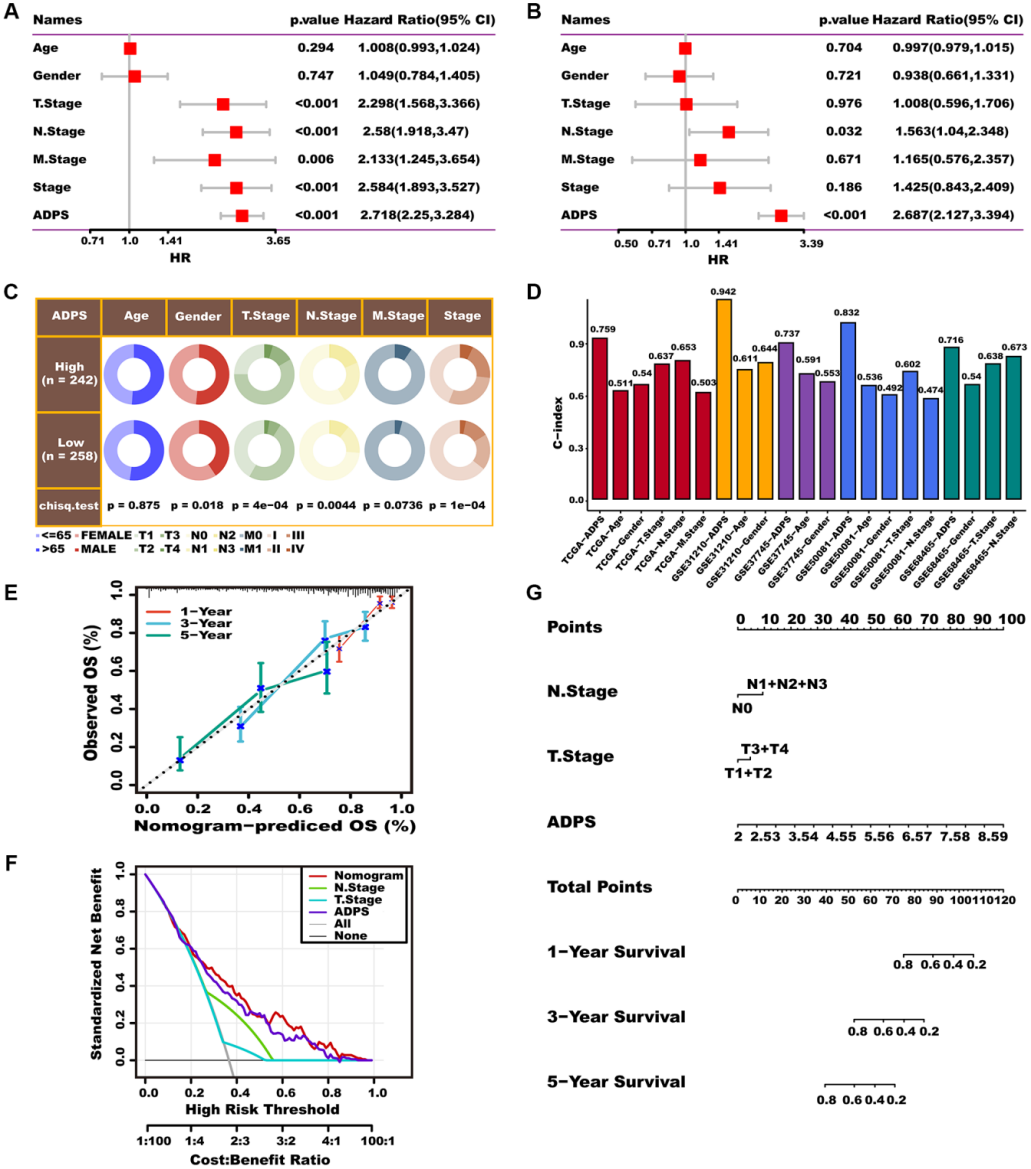
**Prognostic value of the ADPS and nomogram construction.** (**A**, **B**) Univariate Cox (**A**) and multivariate Cox (**B**) analysis of the ADPS and clinicopathological characteristics. (**C**) Circos plot of different clinical factors in two ADPS groups. (**D**) The C-index of the ADPS and various clinical factors in the TCGA, GSE31210, GSE37745, GSE50081, and GSE68465 datasets. (**E**) Calibration curves for 1, 3, and 5 years of nomogram. (**F**) Decision curve for nomogram. (**G**) Nomogram model integrating the ADPS and stage was constructed.

To better apply ADPS in a clinical setting and optimize its predictive performance, we performed univariate and multivariate Cox regression analysis to integrate clinicopathological characteristics as well as ADPS. ADPS was identified as the most significant independent prognostic factor of LUAD according to the results of univariate Cox analysis (hazard ratio (HR) = 2.718, 95% confidence interval (CI): 2.25–3.284, *p* < 0.001), as well as multivariate Cox regression analysis (hazard ration (HR) = 2.687, 95% confidence interval (CI): 2.127–3.394, *p* < 0.001), respectively (Figure 11A, 11B). The circos plot illustrated a significant correlation between ADPS and survival status, tumor stage, as well as TNM staging system in the TCGA cohort (Figure 11C). Moreover, the ADPS showed superior predictive accuracy, as indicated by the higher C-index values, compared to traditional clinical factors including age, gender, tumor stage, and TNM staging system, in various independent LUAD cohorts including TCGA, GSE31210, GSE37745, GSE50081, as well as GSE68465 (Figure 11D). Thus, a nomogram was created by integrating both the stage and ADPS, as illustrated in Figure 11G. The calibration plot illustrated the effective ability of the nomogram in predicting the actual survival outcomes (Figure 11E). Furthermore, the decision curve analysis (DCA) indicated that the nomogram had a superior ability to identify high ADPS patients compared to both the ADPS as well as stage alone, as illustrated in Figure 11F. TimeROC analysis indicated that the ADPS and nomogram had higher AUC values than other indicators (Supplementary Figure 4E). Our research further revealed that ADPS was significantly increased in the T stage (Supplementary Figure 4F), N stage (Supplementary Figure 4G), and tumor stage (Supplementary Figure 4I), without significant differences in M stage (Supplementary Figure 4H).

## DISCUSSION

To date, aggrephagy has escalatingly emerged as a hotspot in anti-tumor research, and several studies have revealed the correlation between aggrephagy modification and the pathogenesis of tumorigenesis [6, 56, 57]. Understanding the mechanisms behind this process could contribute to the development of new treatments for diseases associated with protein aggregation. However, no related study focuses on the potential tumorigenic role of aggrephagy-modified single cells. Multiple algorithms such as AUCell and addmodulescore suggested the entire aggrephagy score was significantly activated in various TME cells in LUAD, and aggrephagy classical genes also shared high heterogeneity among TME. So, comprehensively exploring how aggrephagy shapes and reprograms TME components is necessary. We further dissected the intricate intercellular interactions among aggrephagy-associated TME subtypes in LUAD at the scRNA-seq level and validated the cell types through IHC. This study provides a novel perspective for understanding how the cell-specific expression patterns of aggrephagy genes shape the TME, thus affecting the prognosis and outcomes of immune checkpoint blockade therapy in individual LUAD patients.

CAF, as a highly adaptable and dynamic constituent of the TME, exerts a crucial function in promoting cancer progression via intricate crosstalk with other cellular and non-cellular constituents within the TME [42]. According to their distinct molecular features, CAFs can be categorized into various subtypes, including pan-iCAFs, pan-myCAFs, pan-dCAFs, pan-nCAFs, as well as pan-pCAFs [35]. Until now, there has been no report on the expression patterns of aggrephagy in CAFs. This study identified four subtypes of CAFs: DYNC1I2+CAF-C1, DYNLL1+CAF-C2, PARK7+CAF-C3, and Non-Aggre-CAF-C4, and further explored the interactions between these subtypes and other components in the TME. We found that aggrephagy-related CAFs exhibited more extensive interaction with other components compared to non-aggrephagy-related CAFs. Additionally, we highlighted DYNC1I2+CAF−C1 and PARK7+CAF−C3 because of their high correlation with pan-dCAF, pan-iCAF, and pan-pCAF, along with elevated expression of well-recognized TGFb genes such as TGFB1, SULF1, and THBS2 in DYNC1I2+CAF−C1, as well as pro-inflammatory genes such as IL7, CCL2 and CXCL12 in PARK7+CAF−C3 [58, 59]. We emphasized DYNC1I2+CAF−C1, DYNLL1+CAF−C2, as well as PARK7+CAF−C3 subtypes due to their exceptional ability to differentiate LUAD patient survival. It is worth noting that the high DYNC1I2+CAF−C1, and DYNLL1+CAF−C2 score presented better survival, but the high PARK7+CAF−C3 score presented dismal survival. Thus, we postulated that alterations in aggrephagy could potentially impact the functional and phenotypic properties of CAFs, leading to significant changes in the immunosuppressive TME that may have a consequential impact on the malignant progression and metastasis of LUAD.

Recently, there has been growing interest in studying the role of aggrephagy in immune cell components of the TME, with particular attention focused on TAMs [60]. The TAMs were classified into two subclusters based on NMF clustering, and both subclusters demonstrated broad communication with other TME constituents. The metabolic activity of TAMs has emerged as a critical factor influencing cancer progression and immune responses, with glucose, glutamine, and fatty acid metabolism being among the key metabolic pathways involved [61]. Interestingly, we showed that the two aggrephagy TAM subtypes have distinct metabolic statuses. Obvious activation of pathways such as oxidative phosphorylation, glycolysis/gluconeogenesis, as well as TCA cycle were presented in PARK7+Mac−C2 subtype. We revealed that the functional and metabolic characteristics of TAMs are modulated by aggrephagy genes, pointing towards a potential mechanism of immune evasion facilitated by TAMs in the context of LUAD. In addition, aggrephagy-related subtypes of the four main T cell subtypes demonstrated varying degrees of T cell activity and inactivity. TF analysis revealed that aggrephagy-related subtype all manifested distinct TFs characteristics. For CAFs, TFs such as BHLHE40, FOXO3, ATF3, JUNB, FOSB, as well as CEBPB are notably activated in different aggrephagy-related CAF subtypes. FOSB and JUNB, as constituents of the Activator Protein-1 (AP-1) family, hold a pivotal position in transcriptional control of multiple genes that govern a variety of cellular activities encompassing cell proliferation, differentiation, migration, immune surveillance, and survival [62]. Furthermore, for CD8 T cells, TUBA1A+CD8+ T cells C4 exhibited a unique TF gene signature, such as BATF, BCLAF1, FOSB, and JUNB. Zhang et al. demonstrated that depletion of BATF in diverse chimeric antigen receptor T cell models and mouse OT-1 cells results in enhanced resistance to exhaustion and superior efficacy in tumor eradication [63]. In conclusion, the modulation of distinct transcription factor (TF) regulatory networks by aggrephagy-mediated cell subtypes may result in a reshaped and reprogrammed TME. Furthermore, our cell network analysis demonstrated the close connectivity and communication between these aggrephagy-mediated TME cells and tumor cells. Remarkably, aggrephagy-mediated CAFs and immune cell subtypes exhibited increased crosstalk with cancer epithelial cells, implicating that aggrephagy regulation may contribute to the establishment of an immunosuppressive microenvironment.

Given the intricate intrinsic patterns of aggrephagy in TME cells, we conducted a comprehensive analysis to assess the correlation between the scores of these aggrephagy-related cell subtypes and both prognosis and immune response based on RNA-seq data from multiple centers. Clearly, the degree of aggrephagy gene dominance in TME cells exhibited significant prognostic differences in LUAD patients, and highly distinguished the immune response in patients treated with ICB therapy. These findings highlight the critical role of TME aggrephagy in LUAD, which warrants further investigation. Given the prognostic values of aggrephagy-related cell subtypes, we established a risk model named ADPS with 32 genes. It consisted of 14 protective genes and 18 risk genes. Additionally, ADPS exhibited superior stability in the stratification of patients with different prognoses in multiple cohorts; hence, targeted clinical interventions for patients with varying levels of ADPS are necessary. A lower ADPS was found to be a significant predictor of increased sensitivity to immunotherapy in both the IMvigor210 and GSE91061 cohorts, and this result was further validated by TIDE and Submap analyses.

Furthermore, the comprehensive exploration of immune infiltration from multiple perspectives revealed that the low ADPS group demonstrated a greater abundance of immune cell types, such as activated CD8 T cells and CD4 T cells. It is widely acknowledged that the abundance of effector immune cells, like activated CD8 T cells and CD4 T cells may augment the antitumor immunity and confer better immunotherapeutic outcomes [64, 65]. Consistently, the low ADPS group demonstrated an elevated expression of immune modulators, immune checkpoints, as well as biomarkers that reflect the presence of an immunoreactive microenvironment, like CYT, TCR, and IFN-y. Furthermore, a decreased ADPS was associated with an activated cancer immunity cycle and several immunological pathways, indicating its potential utility in predicting response to immunotherapy. The concept of precision medicine necessitates the early identification of patients who would be responsive to diverse treatments for further personalized interventions. Due to the high sensitivity of the low ADPS patients to immunotherapy, we explored the integration of CTRP, PRISM, as well as CMap databases to create personalized drugs for patients with high ADPS [39, 40, 66]. Finally, BI-2536, a PLK inhibitor, caught our attention. Zhou et al. found BI2536 inhibited lung cancer growth and promoted activation of T cells and DC cells [67]. Going forward, further clinical trials are needed to validate the potential of BI2536 in LUAD, particularly with high ADPS patients. Using a multi-omics approach, we conducted an in-depth investigation into the mutation and copy number variation (CNV) characteristics associated with ADPS. Our findings indicate that the high ADPS group exhibited elevated tumor mutational burden (TMB) and a higher frequency of mutations in the classical tumor suppressor gene TP53 and oncogene KRAS. These mutations have been previously linked to increased invasion and immune evasion in patients with LUAD, leading to a poorer prognosis [68, 69].

After conducting a thorough review of the literature, we discovered that CTBP2 expression was markedly elevated in LUAD tissues when compared to normal lung tissue, and that high levels of CTBP2 expression were linked to a poorer prognosis among LUAD patients [70]. The expression of CCT6A was found to be significantly correlated with both relapse-free and overall survival in patients with LUAD. Moreover, overexpression of CCT6A was observed to enhance cell growth and invasion in LUAD [71]. He et al. proposed a mechanism in which miR-3613-5p expression is induced by RELA through its direct interaction with JUN, thus activating the AKT/mitogen-activated protein kinase (MAPK) pathway and promoting oncogenesis in LUAD [72]. HNRNPF, as a critical alternative splicing regulator, was associated with worse survival of LUAD [73]. DSG2, a member of the cadherin superfamily, has been implicated in cell-cell adhesion and tumorigenesis. Jin et al. demonstrated that high expression of DSG2 is associated with poor prognosis in LUAD patients and promotes cell proliferation and migration, as well as increases resistance to the EGFR tyrosine kinase inhibitor Osimertinib [74]. The overexpression of CACYBP has been shown to enhance the proliferative, invasive, and migratory capacities of LUAD cells, and it may represent a novel therapeutic target for advanced LUAD [75]. Furthermore, our study revealed robust biological associations between ADPS and mutations/TME in LUAD, underscoring the critical role of ADPS in both prognosis and the immune microenvironment. These findings highlight the potential of ADPS as a valuable clinical tool for the precision management and treatment of LUAD.

As an initial investigation, our analysis has some notable limitations including the relatively low sequencing depth of scRNA-seq data and the limited sample size. As a result, further validation in larger patient cohorts is necessary to confirm our conclusions. Compared to bulk RNA-seq, the scRNA-seq analysis of aggrephagy in LUAD is characterized by low coverage and a higher proportion of zero counts, which could potentially introduce bias to the NMF clustering method employed in our study. Additionally, it is regrettable that based on the HPA database, we only found the IHC results for some key aggrephagy-related genes. Further experiments are still required to validate other aggrephagy-related genes, and to further investigate their mechanism of affecting the TME. Quantitative ADPS scoring system included the 32 genes were found to be associated with multiple prognostic features of LUAD, suggesting their potential prognostic value, most of their precise roles in LUAD are still unclear and also require further functional experimental validation in the future.

## CONCLUSIONS

We have identified specific aggrephagy cell subtypes of TME cells for the first time using the single-cell sequencing analysis method. Our findings reveal the aggrephagy-mediated intercellular communication within the tumor microenvironment, which plays a crucial role in regulating tumor growth and modulating the antitumor immune response. Based on machine-learning algorithm, we have developed a robust and powerful signature that accurately predicts the prognosis and immune response of individual LUAD patients, allowing for optimized decision-making and surveillance protocols.

## Supplementary Materials

Supplementary Figures

Supplementary Tables 1, 2, 4, 5 and 8

Supplementary Tables 3, 6, 7 and 9
