# Supplementary material for: Single-cell dissection reveals the role of aggrephagy patterns in tumor microenvironment components aiding predicting prognosis and immunotherapy on lung adenocarcinoma
Source: Aging (Albany NY). 2023 Dec 13;15(23):14333–71. doi: 10.18632/aging.205306 (PMC10756128; doi:10.18632/aging.205306)
Supplement: Supplementary Figures [file aging-15-205306-s001.pdf]

SUPPLEMENTARY FIGURES

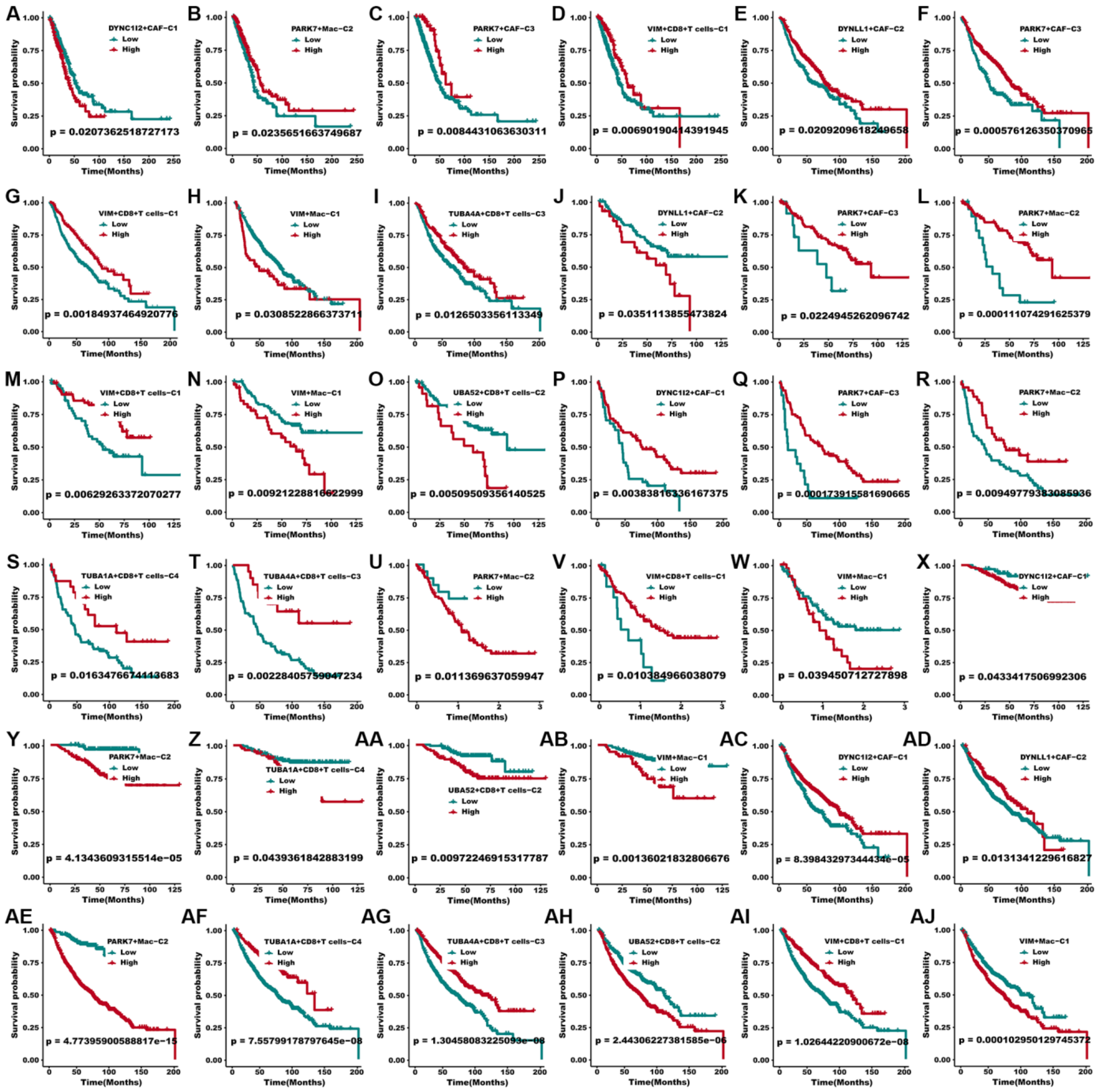

Supplementary Figure 1. Multiple aggrephagy cell subtypes influenced the prognosis of LUAD patients. The K-M curve analysis for aggrephagy-related cell subtypes of DYNC1I2+CAF (**A**), PARK7+Mac (**B**), PARK7+CAF (**C**), and VIM+CD8+T cell (**D**) in the TCGA cohort; DYNLL1+CAF (**E**), PARK7+CAF (**F**), VIM+CD8+T cell (**G**), VIM+Mac (**H**), and TUBA4A+CD8+T cell (**I**) in the GSE68465 cohort; DYNLL1+CAF (**J**), PARK7+CAF (**K**), PARK7+Mac (**L**), VIM+CD8+T cell (**M**), VIM+Mac (**N**), and UBA52+CD8+T cell (**O**) in the GSE50081 cohort; DYNC1I2+CAF (**P**), PARK7+CAF (**Q**), PARK7+Mac (**R**), TUBA1A+CD8+T cell (**S**), and TUBA4A+CD8+T cell (**T**) in the GSE37745 cohort; PARK7+Mac (**U**), VIM+CD8+T cell (**V**), and VIM+Mac (**W**) in the GSE3141 cohort; DYNC1I2+CAF (**X**), PARK7+Mac (**Y**), TUBA1A+CD8+T cell (**Z**), UBA52+CD8+T cell (**AA**), and VIM+Mac (**AB**) in the GSE31210 cohort; DYNC1I2+CAF (**AC**), DYNLL1+CAF (**AD**), PARK7+Mac (**AE**), TUBA1A+CD8+T cell (**AF**), TUBA4A+CD8+T cell (**AG**), UBA52+CD8+T cell (**AH**), VIM+CD8+T cell (**AI**), and VIM+Mac (**AJ**) in the metaGEO cohort.

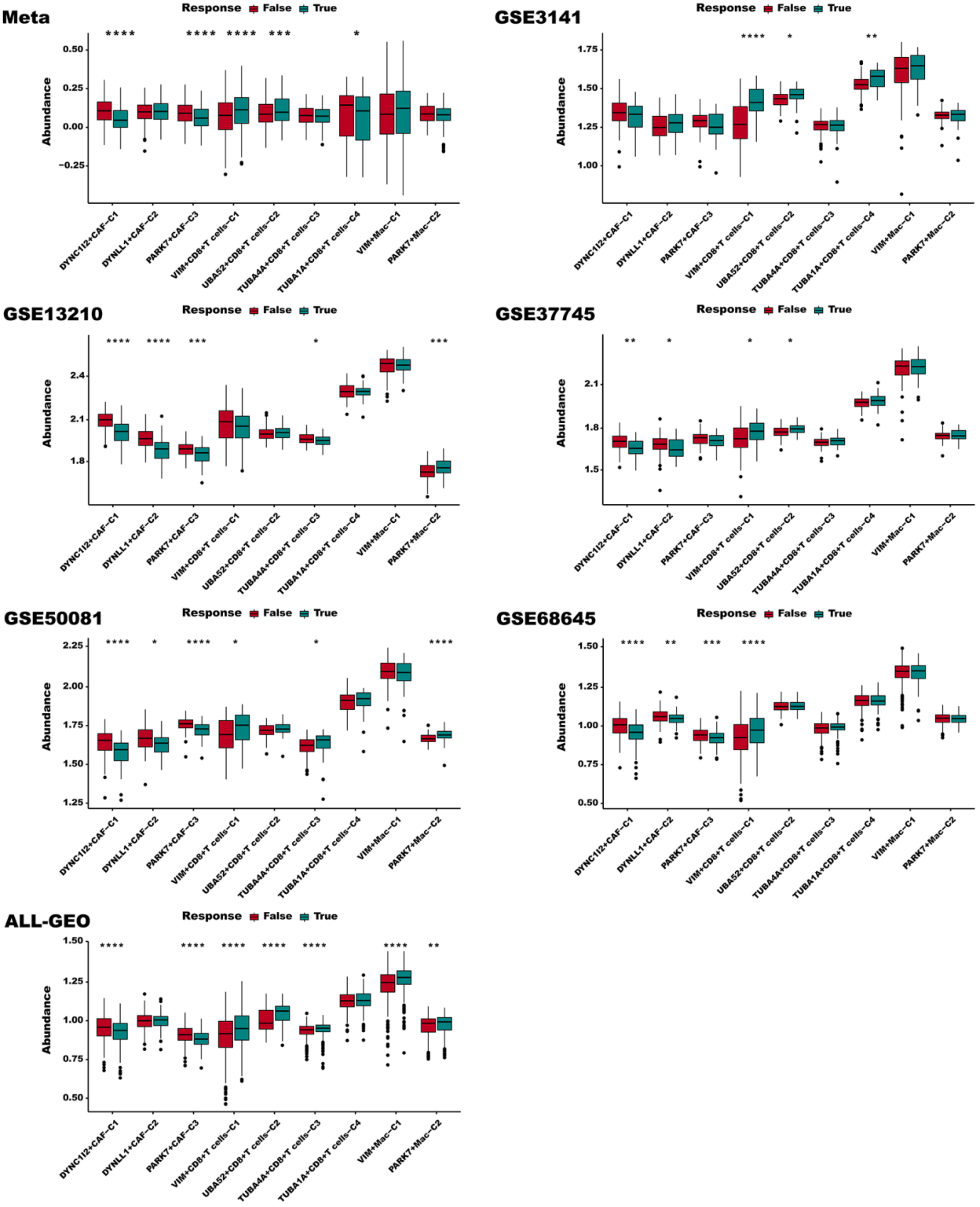

Supplementary Figure 2. Multiple aggrephagy cell subtypes predicted outcomes of immune checkpoint blockade (ICB) therapy. $^{*}p < 0.05$; $^{**}p < 0.01$; $^{***}p < 0.001$. Abbreviation: ns: not significant.

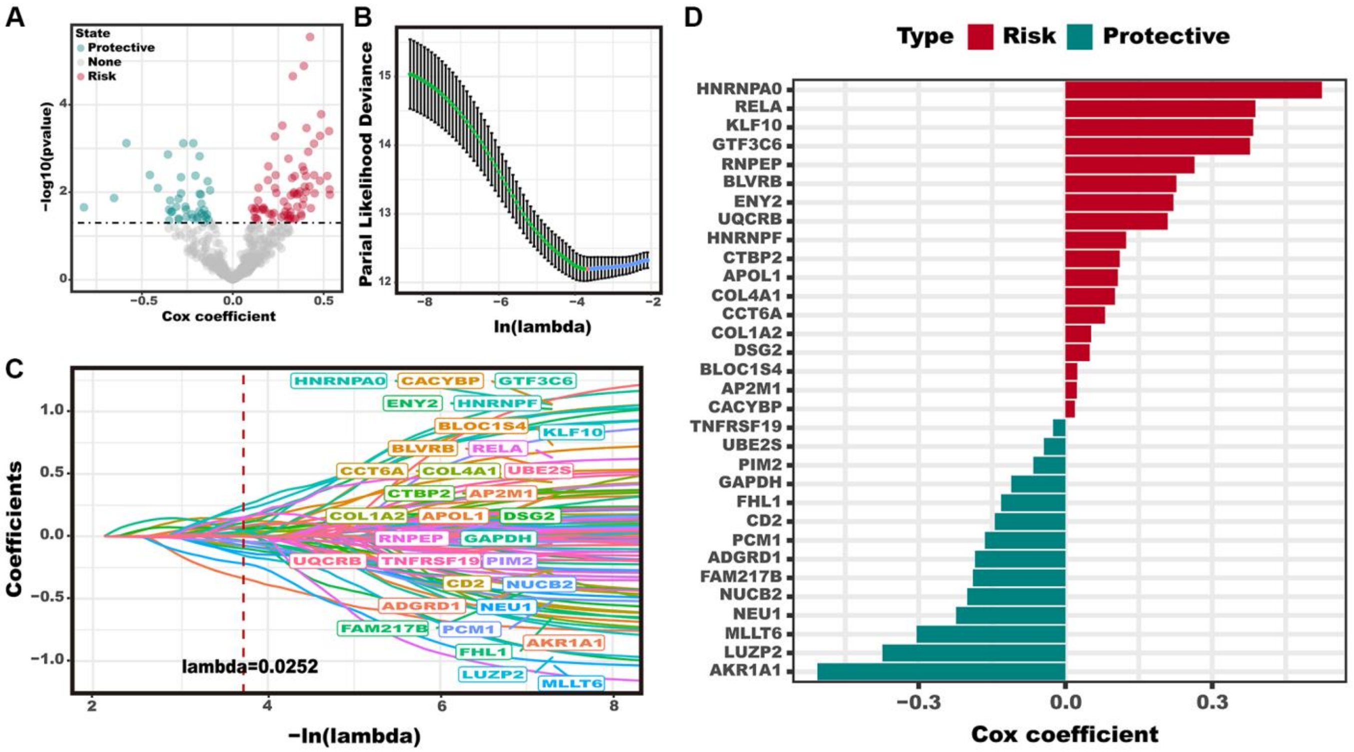

Supplementary Figure 3. The construction of ADPS. (**A**) Volcano plot of prognosis-related genes identified from univariate Cox regression analysis. (**B**) The trajectory of each independent variable with lambda. (**C**) Plots of the produced coefficient distributions for the logarithmic (lambda) series for parameter selection (lambda). (**D**) The multivariate Cox coefficients for each gene in the ADPS.

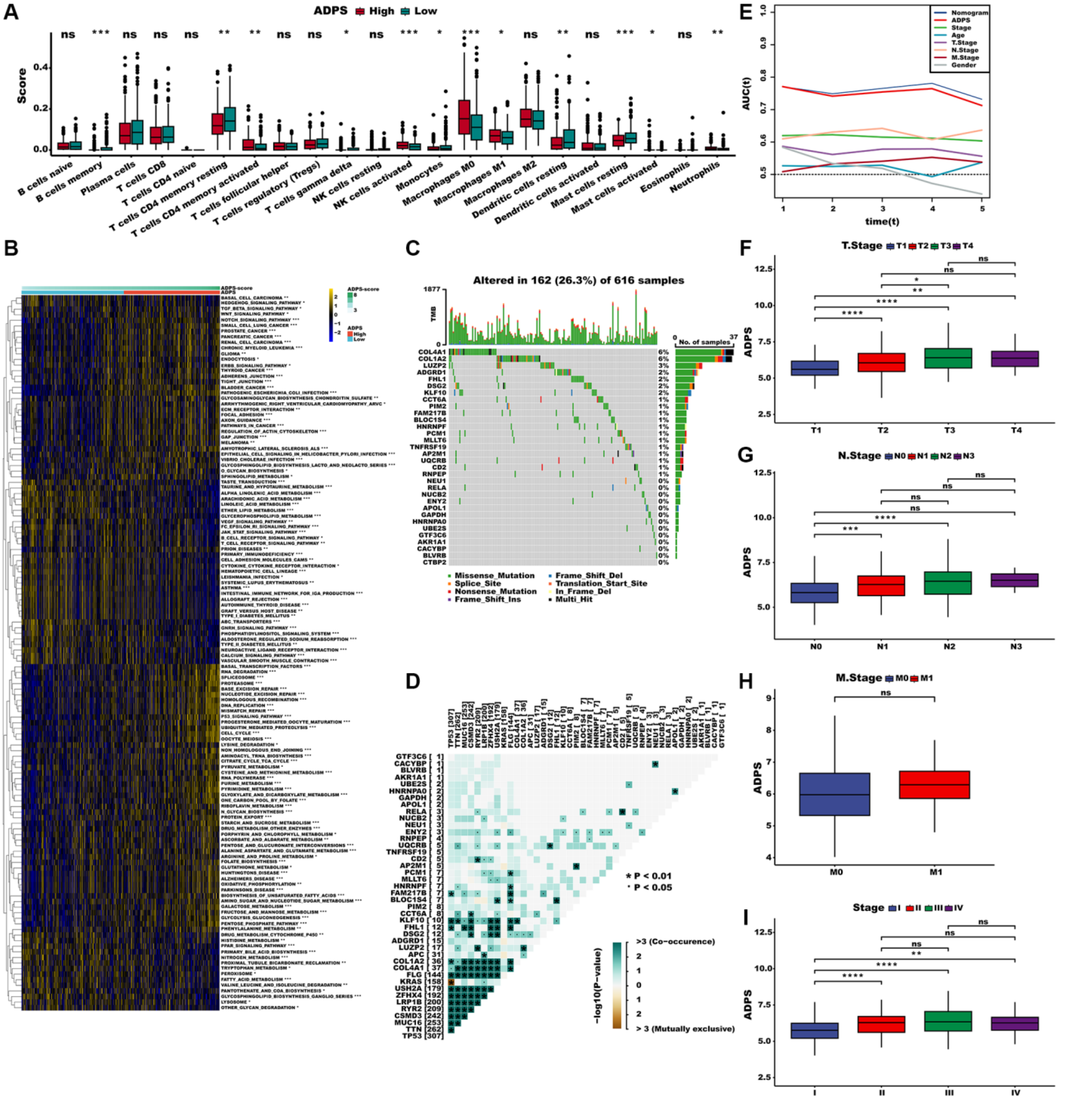

Supplementary Figure 4. Multi-angle comprehensive analysis for ADPS. (**A**) The infiltration abundances of 22 immune cells between high and low ADPS groups using CIBERSORT algorithm. (**B**) KEGG-based GSVA analysis delineated the biological attributes of high and low ADPS groups. (**C**) Waterfall diagram of SNV mutations of ADPS-related genes. (**D**) Collinearity and mutual exclusion analysis of ADPS-related genes and the 10 most mutated genes. (**E**) Comparison of predictive capacity of clinicopathological features and the nomogram using time-ROC analysis. (**F**–**I**) Correlation of clinical characteristics including T stage (**F**), N stage (**G**), M stage (**H**), tumor stage (**I**), and ADPS. $^{*}p < 0.05$; $^{**}p < 0.01$; $^{***}p < 0.001$. Abbreviation: ns: not significant.
