# Supplementary material for: Single-cell dissection reveals the role of aggrephagy patterns in tumor microenvironment components aiding predicting prognosis and immunotherapy on lung adenocarcinoma
Source: Aging (Albany NY). 2023 Dec 13;15(23):14333–71. doi: 10.18632/aging.205306 (PMC10756128; doi:10.18632/aging.205306)
Supplement: Supplementary Tables 3, 6, 7 and 9 [file aging-15-205306-s003.pdf]

SUPPLEMENTARY TABLES

Supplementary Table 3. Feature genes of each NMF cluster in TAMs.

	*p*_val	avg_log2FC	pct.1	pct.2	cluster	gene	Name/Cell type
VIM	0.000211	1.411586	0.595	0.154	0	VIM	VIM+Mac-C1
MT-ND5	0.00067	1.147509	0.952	0.923	0	MT-ND5	VIM+Mac-C1
MT-ND4	0.003345	0.660753	0.952	1	0	MT-ND4	VIM+Mac-C1
MT-ATP6	0.003482	0.668498	0.929	1	0	MT-ATP6	VIM+Mac-C1
EEF2	0.005899	0.673139	0.929	0.808	0	EEF2	VIM+Mac-C1
LYZ	0.006498	0.875906	0.69	0.423	0	LYZ	VIM+Mac-C1
DSG2	0.007983	0.971472	0.238	0	0	DSG2	VIM+Mac-C1
C11orf96	0.009459	0.861673	0.643	0.462	0	C11orf96	VIM+Mac-C1
TOX4	0.009773	1.003141	0.286	0.038	0	TOX4	VIM+Mac-C1
PARK7	1.13E-05	1.208244	0.769	0.333	1	PARK7	PARK7+Mac-C2
DGCR6L	1.96E-05	0.974723	0.5	0.048	1	DGCR6L	PARK7+Mac-C2
TSTD1	0.000156	0.979324	0.692	0.167	1	TSTD1	PARK7+Mac-C2
TMEM9	0.000365	0.785661	0.346	0.024	1	TMEM9	PARK7+Mac-C2
PSMA4	0.000406	0.865812	0.654	0.214	1	PSMA4	PARK7+Mac-C2
PSMB3	0.000409	1.004955	0.538	0.143	1	PSMB3	PARK7+Mac-C2
SMIM4	0.000426	0.763388	0.577	0.143	1	SMIM4	PARK7+Mac-C2
CFAP298	0.000456	0.719377	0.269	0	1	CFAP298	PARK7+Mac-C2
PSENEN	0.000984	0.581205	0.385	0.048	1	PSENEN	PARK7+Mac-C2
CMTM6	0.001005	0.774987	0.308	0.024	1	CMTM6	PARK7+Mac-C2
MCUR1	0.001084	0.560481	0.308	0.024	1	MCUR1	PARK7+Mac-C2
TUBA4A	0.001124	1.001012	0.346	0.048	1	TUBA4A	PARK7+Mac-C2
FH	0.001169	0.570502	0.308	0.024	1	FH	PARK7+Mac-C2
NARS2	0.001286	0.700944	0.231	0	1	NARS2	PARK7+Mac-C2
CRTAP	0.001286	0.68988	0.231	0	1	CRTAP	PARK7+Mac-C2
DYNLL2	0.001286	0.654695	0.231	0	1	DYNLL2	PARK7+Mac-C2
TMEM92	0.001286	0.53154	0.231	0	1	TMEM92	PARK7+Mac-C2
PPIE	0.001693	0.693616	0.346	0.048	1	PPIE	PARK7+Mac-C2
PSMC4	0.001693	0.616486	0.346	0.048	1	PSMC4	PARK7+Mac-C2
NRGN	0.001901	0.577685	0.5	0.119	1	NRGN	PARK7+Mac-C2
YWHAQ	0.001942	0.599586	0.654	0.238	1	YWHAQ	PARK7+Mac-C2
SEMA4A	0.001944	0.820897	0.385	0.071	1	SEMA4A	PARK7+Mac-C2
LSM4	0.002023	0.843285	0.423	0.095	1	LSM4	PARK7+Mac-C2
QKI	0.00216	0.687433	0.269	0.024	1	QKI	PARK7+Mac-C2
SLC35C2	0.00216	0.658456	0.269	0.024	1	SLC35C2	PARK7+Mac-C2
SMIM19	0.002199	0.628397	0.385	0.071	1	SMIM19	PARK7+Mac-C2
DNPH1	0.002358	0.531032	0.346	0.048	1	DNPH1	PARK7+Mac-C2
HIST1H1C	0.002408	1.004697	0.423	0.095	1	HIST1H1C	PARK7+Mac-C2
CALM2	0.002578	0.654472	1	0.69	1	CALM2	PARK7+Mac-C2
SERINC3	0.002691	0.757213	0.308	0.048	1	SERINC3	PARK7+Mac-C2
NUCB2	0.002891	0.682196	0.462	0.119	1	NUCB2	PARK7+Mac-C2
GJA1	0.00291	0.67725	0.269	0.024	1	GJA1	PARK7+Mac-C2
MTIF3	0.00291	0.572991	0.269	0.024	1	MTIF3	PARK7+Mac-C2
DESI2	0.002979	0.559832	0.385	0.071	1	DESI2	PARK7+Mac-C2
NDUFB2	0.003046	0.747608	0.692	0.262	1	NDUFB2	PARK7+Mac-C2
CDK4	0.003131	0.620115	0.269	0.024	1	CDK4	PARK7+Mac-C2
CYC1	0.003243	0.797487	0.346	0.071	1	CYC1	PARK7+Mac-C2
NDUFA4	0.003264	0.769977	0.846	0.476	1	NDUFA4	PARK7+Mac-C2
UQCRB	0.003271	0.691112	0.923	0.595	1	UQCRB	PARK7+Mac-C2
GPRC5C	0.003287	0.632321	0.308	0.048	1	GPRC5C	PARK7+Mac-C2
C21orf2	0.003368	0.501911	0.269	0.024	1	C21orf2	PARK7+Mac-C2
RAB11B	0.003479	0.521213	0.538	0.167	1	RAB11B	PARK7+Mac-C2
PLPP5	0.003559	0.655067	0.385	0.071	1	PLPP5	PARK7+Mac-C2

TIMM10B	0.003567	0.658733	0.192	0	1	TIMM10B	PARK7+Mac-C2
MORN2	0.003567	0.601826	0.192	0	1	MORN2	PARK7+Mac-C2
UBLCP1	0.003567	0.551008	0.192	0	1	UBLCP1	PARK7+Mac-C2
TMEM129	0.003567	0.504385	0.192	0	1	TMEM129	PARK7+Mac-C2
RAN	0.003633	0.719248	0.615	0.238	1	RAN	PARK7+Mac-C2
EIF3F	0.003655	0.569545	0.538	0.167	1	EIF3F	PARK7+Mac-C2
TAOK3	0.003748	0.654915	0.308	0.048	1	TAOK3	PARK7+Mac-C2
RAP1A	0.004	0.606681	0.423	0.095	1	RAP1A	PARK7+Mac-C2
HMGCL	0.004001	0.526426	0.308	0.048	1	HMGCL	PARK7+Mac-C2
ATXN2	0.004268	0.657027	0.308	0.048	1	ATXN2	PARK7+Mac-C2
EIF5A	0.004314	0.643102	0.692	0.286	1	EIF5A	PARK7+Mac-C2
HADHB	0.004643	0.577399	0.385	0.095	1	HADHB	PARK7+Mac-C2
PFN1	0.004726	0.704733	0.846	0.548	1	PFN1	PARK7+Mac-C2
HMOX1	0.004852	0.508662	0.308	0.048	1	HMOX1	PARK7+Mac-C2
MRPL42	0.004942	0.606266	0.346	0.071	1	MRPL42	PARK7+Mac-C2
CDK2AP2	0.005013	0.962554	0.577	0.286	1	CDK2AP2	PARK7+Mac-C2
MAGED1	0.005241	0.534179	0.346	0.071	1	MAGED1	PARK7+Mac-C2
P3H2	0.005377	0.67345	0.5	0.143	1	P3H2	PARK7+Mac-C2
MLF2	0.005384	0.567608	0.538	0.167	1	MLF2	PARK7+Mac-C2
ARPC3	0.005721	0.589591	0.615	0.262	1	ARPC3	PARK7+Mac-C2
PSMA5	0.005791	0.563824	0.385	0.095	1	PSMA5	PARK7+Mac-C2
HDDC2	0.006115	0.58931	0.385	0.095	1	HDDC2	PARK7+Mac-C2
NT5DC1	0.006149	0.649745	0.231	0.024	1	NT5DC1	PARK7+Mac-C2
ADGRD1	0.006149	0.647112	0.231	0.024	1	ADGRD1	PARK7+Mac-C2
CAPRIN1	0.006611	0.608691	0.231	0.024	1	CAPRIN1	PARK7+Mac-C2
MT1X	0.006617	0.872285	0.423	0.119	1	MT1X	PARK7+Mac-C2
ENY2	0.0067	0.584781	0.5	0.167	1	ENY2	PARK7+Mac-C2
ANXA7	0.006791	0.748266	0.462	0.143	1	ANXA7	PARK7+Mac-C2
ZNF593	0.006964	0.519489	0.423	0.119	1	ZNF593	PARK7+Mac-C2
HIP1R	0.007105	0.550525	0.231	0.024	1	HIP1R	PARK7+Mac-C2
MRPS26	0.007215	0.622555	0.308	0.071	1	MRPS26	PARK7+Mac-C2
MRPL52	0.007583	0.58825	0.385	0.095	1	MRPL52	PARK7+Mac-C2
CIB1	0.007611	0.716517	0.615	0.31	1	CIB1	PARK7+Mac-C2
GCN1	0.007631	0.568252	0.231	0.024	1	GCN1	PARK7+Mac-C2
SNF8	0.007631	0.506375	0.231	0.024	1	SNF8	PARK7+Mac-C2
ME2	0.007631	0.503717	0.231	0.024	1	ME2	PARK7+Mac-C2
SELENOM	0.008263	0.670829	0.654	0.381	1	SELENOM	PARK7+Mac-C2
ST3GAL5	0.008292	0.575376	0.462	0.167	1	ST3GAL5	PARK7+Mac-C2
COX7A2	0.008756	0.618256	0.731	0.381	1	COX7A2	PARK7+Mac-C2
BLVRB	0.0088	0.681885	0.615	0.238	1	BLVRB	PARK7+Mac-C2
CTSD	0.00922	0.731674	0.808	0.452	1	CTSD	PARK7+Mac-C2
IFT57	0.009459	0.782298	0.769	0.452	1	IFT57	PARK7+Mac-C2
IGF2R	0.009644	0.704461	0.308	0.071	1	IGF2R	PARK7+Mac-C2
CHCHD2	0.009661	0.525212	0.846	0.524	1	CHCHD2	PARK7+Mac-C2
UBE2S	0.009769	0.59751	0.154	0	1	UBE2S	PARK7+Mac-C2
CHMP7	0.009769	0.521948	0.154	0	1	CHMP7	PARK7+Mac-C2
PIGF	0.009769	0.520695	0.154	0	1	PIGF	PARK7+Mac-C2
TRPT1	0.009769	0.507902	0.154	0	1	TRPT1	PARK7+Mac-C2
CGGBP1	0.009773	0.61168	0.346	0.095	1	CGGBP1	PARK7+Mac-C2
FNIP1	0.009823	0.619406	0.269	0.048	1	FNIP1	PARK7+Mac-C2
ZNF511	0.009823	0.590366	0.269	0.048	1	ZNF511	PARK7+Mac-C2
PSMA2	0.009857	0.590351	0.385	0.095	1	PSMA2	PARK7+Mac-C2
MGLL	0.009864	0.558292	0.615	0.286	1	MGLL	PARK7+Mac-C2

Supplementary Table 6. Univariate Cox regression analysis in the TCGA, GSE3141, GSE3141, GSE37745, GSE50081, GSE68465, MetaGEO, and Meta cohorts.

TCGA				
**id**	**HR**	**HR.95L**	**HR.95H**	***p*-value**
DYNC1I2+CAF-C1	2.581799054	0.604868	11.02007	0.200201
DYNLL1+CAF-C2	4.077003236	0.404556	41.08693	0.233168
PARK7+CAF-C3	0.802670531	0.104466	6.167338	0.832667
VIM+CD8+T_cells-C1	0.183536583	0.051212	0.657767	0.009236
UBA52+CD8+T_cells-C2	3.037373724	0.253424	36.40397	0.380636
TUBA4A+CD8+T_cells-C3	2.073828361	0.069197	62.15291	0.674162
TUBA1A+CD8+T_cells-C4	2.275863571	0.255933	20.23791	0.460759
VIM+Mac-C1	1.63499456	0.235508	11.35083	0.618977
PARK7+Mac-C2	0.875312247	0.107903	7.100527	0.90077
**GSE3141**				
**id**	**HR**	**HR.95L**	**HR.95H**	***p*-value**
DYNC1I2+CAF-C1	2.007242198	0.16733	24.07832	0.582561
DYNLL1+CAF-C2	0.968835927	0.049885	18.81621	0.983311
PARK7+CAF-C3	1.956635837	0.080021	47.84294	0.680674
VIM+CD8+T_cells-C1	0.232823649	0.040624	1.334361	0.101808
UBA52+CD8+T_cells-C2	0.858300774	0.009532	77.28693	0.946942
TUBA4A+CD8+T_cells-C3	0.813117539	0.010904	60.63317	0.925077
TUBA1A+CD8+T_cells-C4	0.315129975	0.008938	11.11082	0.525245
VIM+Mac-C1	0.823288349	0.157964	4.29087	0.817434
PARK7+Mac-C2	12.6988644	0.041296	3905.024	0.384542
**GSE31210**				
**id**	**HR**	**HR.95L**	**HR.95H**	***p*-value**
DYNC1I2+CAF-C1	20.1376868	0.21446	1890.92	0.195108
DYNLL1+CAF-C2	2.927174004	0.049746	172.2432	0.605436
PARK7+CAF-C3	3.486241245	0.009597	1266.455	0.677998
VIM+CD8+T_cells-C1	1.431754564	0.074284	27.59557	0.812078
UBA52+CD8+T_cells-C2	769.3726441	0.576163	1027372	0.070325
TUBA4A+CD8+T_cells-C3	44.61804366	0.007434	267805.8	0.392181
TUBA1A+CD8+T_cells-C4	214.9467924	0.189983	243191.3	0.134392
VIM+Mac-C1	1610.479881	2.262259	1146485	0.027554
PARK7+Mac-C2	15455.07325	37.59304	6353817	0.001684
**GSE37745**				
**id**	**HR**	**HR.95L**	**HR.95H**	***p*-value**
DYNC1I2+CAF-C1	0.128390992	0.003231	5.101943	0.274582
DYNLL1+CAF-C2	0.133755798	0.004667	3.833287	0.239963
PARK7+CAF-C3	0.009723816	8.47E-05	1.11609	0.055546
VIM+CD8+T_cells-C1	0.382683171	0.044922	3.259984	0.379507
UBA52+CD8+T_cells-C2	0.198820187	0.000245	161.6298	0.636573
TUBA4A+CD8+T_cells-C3	0.000288953	9.14E-07	0.091323	0.005521
TUBA1A+CD8+T_cells-C4	0.003347176	2.15E-05	0.520577	0.026864
VIM+Mac-C1	0.248788483	0.022933	2.699033	0.252752
PARK7+Mac-C2	0.000769389	1.10E-06	0.537409	0.031888

		**GSE50081**		
**id**	**HR**	**HR.95L**	**HR.95H**	***p*-value**
DYNC1I2+CAF-C1	0.737622411	0.036687	14.83047	0.842455
DYNLL1+CAF-C2	0.532903037	0.015622	18.17822	0.726709
PARK7+CAF-C3	0.011703745	4.57E-05	2.998346	0.115973
VIM+CD8+T_cells-C1	0.075712218	0.007643	0.750048	0.027399
UBA52+CD8+T_cells-C2	4.950905497	0.004651	5270.491	0.65287
TUBA4A+CD8+T_cells-C3	0.107274024	0.00327	3.519127	0.210032
TUBA1A+CD8+T_cells-C4	0.225455696	0.004102	12.39032	0.466177
VIM+Mac-C1	0.917315219	0.03395	24.78576	0.959077
PARK7+Mac-C2	2.65E-05	3.01E-08	0.02332	0.002317
		**GSE68465**		
**id**	**HR**	**HR.95L**	**HR.95H**	***p*-value**
DYNC1I2+CAF-C1	0.724513433	0.135067	3.886377	0.706904
DYNLL1+CAF-C2	0.228289121	0.012141	4.292474	0.323763
PARK7+CAF-C3	0.039698618	0.003022	0.521497	0.014071
VIM+CD8+T_cells-C1	0.203946722	0.072489	0.573797	0.002591
UBA52+CD8+T_cells-C2	0.477837403	0.013018	17.53924	0.687883
TUBA4A+CD8+T_cells-C3	0.183298861	0.013166	2.551969	0.206694
TUBA1A+CD8+T_cells-C4	1.211091094	0.103747	14.13772	0.878589
VIM+Mac-C1	3.310688928	0.503132	21.78487	0.212989
PARK7+Mac-C2	0.239756992	0.00661	8.695918	0.4357
		**MetaGEO**		
**id**	**HR**	**HR.95L**	**HR.95H**	***p*-value**
DYNC1I2+CAF-C1	0.152365738	0.049322	0.470686	0.001078
DYNLL1+CAF-C2	0.14848377	0.022511	0.979421	0.047528
PARK7+CAF-C3	0.825739866	0.153063	4.45469	0.823797
VIM+CD8+T_cells-C1	0.079643086	0.03838	0.165268	1.10E-11
UBA52+CD8+T_cells-C2	10.40697494	2.854632	37.94014	0.000386
TUBA4A+CD8+T_cells-C3	0.009589995	0.001341	0.068575	3.66E-06
TUBA1A+CD8+T_cells-C4	0.020902299	0.00466	0.093757	4.39E-07
VIM+Mac-C1	2.372205799	0.762971	7.37559	0.135561
PARK7+Mac-C2	368.5834853	76.27593	1781.083	1.94E-13
		**Meta**		
**id**	**HR**	**HR.95L**	**HR.95H**	***p*-value**
DYNC1I2+CAF-C1	0.319938207	0.132078	0.774998	0.011582
DYNLL1+CAF-C2	0.238401367	0.047903	1.186461	0.079921
PARK7+CAF-C3	2.963187473	1.006161	8.726711	0.048711
VIM+CD8+T_cells-C1	0.096224073	0.051225	0.180753	3.39E-13
UBA52+CD8+T_cells-C2	13.37013396	4.244167	42.1191	9.47E-06
TUBA4A+CD8+T_cells-C3	0.015943941	0.003321	0.076536	2.33E-07
TUBA1A+CD8+T_cells-C4	0.163465178	0.082465	0.324029	2.13E-07
VIM+Mac-C1	4.621473744	1.864315	11.45623	0.000951
PARK7+Mac-C2	56.51273294	16.12425	198.0675	2.88E-10

Supplementary Table 7. Logistic regression analysis in the TCGA, GSE3141, GSE3141, GSE37745, GSE50081, GSE68465, MetaGEO, and Meta cohorts.

TCGA				
**gene**	**OR**	**OR.95L**	**OR.95H**	***p*-value**
DYNC1I2+CAF-C1	2.02E-05	1.56E-06	0.000222941	1.26E-17
DYNLL1+CAF-C2	0.000147	4.94E-06	0.003793619	1.84E-07
PARK7+CAF-C3	0.001265	4.42E-05	0.0272173	4.56E-05
VIM+CD8+T_cells-C1	40.32358	7.557707	229.156878	2.10E-05
UBA52+CD8+T_cells-C2	3.943248	0.172431	98.16945486	0.394881
TUBA4A+CD8+T_cells-C3	0.008325	9.72E-05	0.671910613	0.033252
TUBA1A+CD8+T_cells-C4	4.518784	0.251759	86.37529831	0.309754
VIM+Mac-C1	0.865458	0.067049	11.55283268	0.912121
PARK7+Mac-C2	54.95842	2.77556	1353.95107	0.011093
**GSE3141**				
**gene**	**OR**	**OR.95L**	**OR.95H**	***p*-value**
DYNC1I2+CAF-C1	0.066885	0.001432	2.721712047	0.155861
DYNLL1+CAF-C2	8.904625	0.095688	939.4348189	0.347523
PARK7+CAF-C3	0.027254	0.000181	3.088246493	0.141055
VIM+CD8+T_cells-C1	4476.675	123.5161	308868.5754	2.20E-05
UBA52+CD8+T_cells-C2	621.5397	0.656213	1128245.268	0.077061
TUBA4A+CD8+T_cells-C3	0.566985	0.001741	238.2310752	0.846678
TUBA1A+CD8+T_cells-C4	8573.647	21.15131	6205252.904	0.004536
VIM+Mac-C1	6.641665	0.483144	140.100899	0.186218
PARK7+Mac-C2	0.145823	9.51E-05	222.6762122	0.595843
**GSE31210**				
**gene**	**OR**	**OR.95L**	**OR.95H**	***p*-value**
DYNC1I2+CAF-C1	1.57E-08	5.52E-11	2.30E-06	3.09E-11
DYNLL1+CAF-C2	8.33E-07	6.41E-09	6.45E-05	2.29E-09
PARK7+CAF-C3	5.54E-05	2.63E-07	0.008595146	0.000208
VIM+CD8+T_cells-C1	0.230344	0.01945	2.626560295	0.238935
UBA52+CD8+T_cells-C2	18.13677	0.047548	7346.64122	0.339587
TUBA4A+CD8+T_cells-C3	9.01E-05	4.59E-08	0.130091197	0.013627
TUBA1A+CD8+T_cells-C4	1.215349	0.003995	380.510701	0.946583
VIM+Mac-C1	1.68501	0.020167	157.2439218	0.818457
PARK7+Mac-C2	11112.94	81.65885	2129205.262	0.000313
**GSE37745**				
**gene**	**OR**	**OR.95L**	**OR.95H**	***p*-value**
DYNC1I2+CAF-C1	1.56E-05	9.64E-09	0.011584066	0.001776
DYNLL1+CAF-C2	0.011445	2.63E-05	3.352479758	0.131502
PARK7+CAF-C3	0.001902	7.41E-07	3.301046233	0.105386
VIM+CD8+T_cells-C1	233.1595	3.44978	25464.279	0.015854
UBA52+CD8+T_cells-C2	1329463	13.95029	4.08834E+11	0.020925
TUBA4A+CD8+T_cells-C3	995.7538	0.017683	121548210.4	0.227426

TUBA1A+CD8+T_cells-C4	440.6525	0.062058	5707936.256	0.188809
VIM+Mac-C1	3.665756	0.064324	314.2394703	0.541082
PARK7+Mac-C2	15.80532	0.000352	1051811.376	0.615967
**GSE50081**				
**gene**	**OR**	**OR.95L**	**OR.95H**	***p*-value**
DYNC1I2+CAF-C1	0.000681	7.33E-06	0.03809135	0.000771
DYNLL1+CAF-C2	0.005953	5.15E-05	0.511578096	0.02799
PARK7+CAF-C3	1.62E-07	1.97E-11	0.000440894	0.000274
VIM+CD8+T_cells-C1	55.8003	2.057267	1976.221672	0.020897
UBA52+CD8+T_cells-C2	290.5348	0.066782	2378699.731	0.197526
TUBA4A+CD8+T_cells-C3	13.8598	0.123247	2421.284465	0.292375
TUBA1A+CD8+T_cells-C4	7.610202	0.049434	1659.706252	0.440633
VIM+Mac-C1	0.120726	0.002691	4.754539676	0.259671
PARK7+Mac-C2	51058593	1595.171	6.24062E+12	0.001531
**GSE68465**				
**gene**	**OR**	**OR.95L**	**OR.95H**	***p*-value**
DYNC1I2+CAF-C1	0.000139	6.99E-06	0.002332162	1.95E-09
DYNLL1+CAF-C2	0.001069	1.22E-05	0.082101774	0.002288
PARK7+CAF-C3	0.000621	1.06E-05	0.032392375	0.000302
VIM+CD8+T_cells-C1	39.02222	7.384114	221.9286704	2.35E-05
UBA52+CD8+T_cells-C2	10.77913	0.055413	2186.920845	0.377501
TUBA4A+CD8+T_cells-C3	51.31209	0.942561	3272.378611	0.05756
TUBA1A+CD8+T_cells-C4	2.662463	0.07605	98.85240086	0.591231
VIM+Mac-C1	2.441731	0.179217	35.77694955	0.507038
PARK7+Mac-C2	0.34741	0.001827	66.31239484	0.692391
**MetaGEO**				
**gene**	**OR**	**OR.95L**	**OR.95H**	***p*-value**
DYNC1I2+CAF-C1	0.012352	0.002438	0.060578191	8.02E-08
DYNLL1+CAF-C2	1.696705	0.138144	21.00039256	0.679659
PARK7+CAF-C3	5.38E-05	4.30E-06	0.000624731	9.39E-15
VIM+CD8+T_cells-C1	28.65448	9.698947	87.3198209	2.10E-09
UBA52+CD8+T_cells-C2	18280.18	2659.294	132406.6492	6.83E-23
TUBA4A+CD8+T_cells-C3	89.62167	5.018813	1742.524109	0.002559
TUBA1A+CD8+T_cells-C4	10.70487	1.327287	88.44545704	0.026751
VIM+Mac-C1	128.4665	27.19828	648.9844333	1.93E-09
PARK7+Mac-C2	13.64775	2.197253	87.68294855	0.005405
**Meta**				
**gene**	**OR**	**OR.95L**	**OR.95H**	***p*-value**
DYNC1I2+CAF-C1	0.000153	3.48E-05	0.000648301	5.30E-32
DYNLL1+CAF-C2	1.025265	0.217904	4.826020841	0.974798
PARK7+CAF-C3	0.002304	0.000485	0.010668186	1.29E-14
VIM+CD8+T_cells-C1	10.81759	4.553453	26.00137186	8.31E-08
UBA52+CD8+T_cells-C2	12.15326	3.784088	39.39289892	2.90E-05
TUBA4A+CD8+T_cells-C3	0.509344	0.086712	2.983289218	0.454582
TUBA1A+CD8+T_cells-C4	0.42945	0.221176	0.832975222	0.01243

VIM+Mac-C1	1.318582	0.750752	2.319263204	0.33623
PARK7+Mac-C2	0.206939	0.035085	1.211979935	0.081093

Supplementary Table 9. The ADPS-related genes and corresponding coefficients.

Gene	Coef	Type
LUZP2	−0.374121637	Protective
COL4A1	0.101170202	Risk
GTF3C6	0.377254728	Risk
CTBP2	0.110815334	Risk
AKR1A1	−0.506961197	Protective
COL1A2	0.052344441	Risk
TNFRSF19	−0.025303508	Protective
APOL1	0.107021888	Risk
AP2M1	0.023375955	Risk
KLF10	0.383501703	Risk
FHL1	−0.131528	Protective
FAM217B	−0.189208623	Protective
BLOC1S4	0.024211454	Risk
UQCRB	0.208941759	Risk
HNRNPF	0.124065381	Risk
NEU1	−0.223584911	Protective
CCT6A	0.080899978	Risk
RNPEP	0.263793282	Risk
GAPDH	−0.110872233	Protective
RELA	0.388110159	Risk
NUCB2	−0.200828737	Protective
PCM1	−0.164689427	Protective
MLLT6	−0.304064015	Protective
CD2	−0.144521072	Protective
PIM2	−0.065727646	Protective
HNRNPA0	0.524133487	Risk
CACYBP	0.019093532	Risk
DSG2	0.049422674	Risk
ADGRD1	−0.18487887	Protective
ENY2	0.220663515	Risk
BLVRB	0.226676186	Risk
UBE2S	−0.044076029	Protective
